# Part II—Volatile Profiles of Kiwi Kefir-like Beverages Influenced by the Amount of Inoculum, Shaking Rate, and Successive Kefir Grain Passages

**DOI:** 10.3390/foods14142502

**Published:** 2025-07-17

**Authors:** Delicia L. Bazán, Sandra Cortés Diéguez, José Manuel Domínguez, Nelson Pérez-Guerra

**Affiliations:** 1 Departamento Académico de Ingeniería de Industrias Alimentarias, Facultad de Ingeniería, Universidad Nacional de Jaén, Carretera Jaén–San Ignacio KM 24–Sect., Yanuyacu, Jaén 06801, Peru; delicia.bazan@unj.edu.pe; 2 Industrial Biotechnology and Environmental Engineering Group “BiotecnIA”, Department of Analytical and Food Chemistry, University of Vigo, 32004 Ourense, Spain; 3 Industrial Biotechnology and Environmental Engineering Group “BiotecnIA”, Chemical Engineering Department, University of Vigo, 32004 Ourense, Spain; smcortes@uvigo.gal (S.C.D.); jmanuel@uvigo.gal (J.M.D.)

**Keywords:** kiwi kefir–like beverage, proportions of milk kefir grains, shaking rate, volatile profile, cluster analysis, principal component analysis, response surface methodology

## Abstract

This study analyzes the aromatic profiles of kiwi-based fermented beverages, inoculated with varying proportions of milk kefir grains and incubated under different shaking rates. The experiments were designed using response surface methodology and three consecutive batch cultures were performed under each experimental condition. At the end of each fermentation, the grains were separated from the beverage and reused as the inoculum for fermenting fresh kiwi juice in the subsequent batch. Based on the results, together with the previously determined microbiological and chemical characteristics, two beverages were identified as having broader aromatic profiles, lower contents of sugars, ethanol, and acids, and high counts of lactic acid bacteria (LAB) and yeasts (>10^6^ CFU/mL). These beverages were produced under relatively low agitation rates (38 and 86 rpm) and high inoculum proportions (4.33% and 4.68% *w*/*v*) during the second and third batch cultures, respectively. Over 28 days of refrigerated storage, the pH values of both beverages remained relatively stable, and the LAB counts consistently exceeded 10^6^ CFU/mL. Yeast counts, along with the production of ethanol, glycerol, lactic acid, and acetic acid, increased slightly over time. In contrast, the concentrations of citric acid, quinic acid, total sugars, and acetic acid bacteria declined by day 28.

## 1. Introduction

Fruit juices commonly contain high levels of monosaccharides (mainly glucose and fructose), which can contribute significantly to a high caloric intake for consumers. An effective and cost-efficient strategy to reduce this sugar content involves fermenting the juices using either single cultures of probiotic strains [1,2,3,4,5,6] or kefir grains [7,8,9]. This process not only reduces the sugar content but also yields a potentially probiotic fermented beverage with a pleasant aromatic profile. Additionally, this approach may support the valorization of fruits with a low commercial value (such as undersized fruit) or those resulting from surplus production.

In recent years, the development of fruit-based kefir beverages [7,8,9,10,11,12,13] has been explored as an alternative to traditional milk kefir, which contains lactose and may not be suitable for lactose-intolerant individuals. These beverages have been shown to contain high levels of lactic acid bacteria (LAB) and yeasts, often exceeding 10^6^ CFU/mL, the minimum threshold required to confer probiotic benefits to the consumer. Furthermore, fruit-based kefir beverages are rich in volatile compounds, derived both from the original fruit juice and from microbial activity during fermentation by organisms present in kefir grains [9,10,11,12,13] or kefir-based inocula [7,8].

Several studies have shown that both the shaking rate [11,14,15] and the amount of kefir grains [11,14,16,17,18] can influence the nutrient consumption, kefir grain growth, viable cell production, and synthesis of fermentation metabolites during the fermentation of various dairy and non-dairy substrates. However, the results have been controversial, as the optimal values for the shaking rate and proportions of milk kefir grains varied for different types of beverages.

For example, the growth rate of kefir grains was maximal under static conditions in milk [14] or at 125 rpm in skim milk [15].

Within an inoculum range of 1% to 5% (*w*/*v*), the highest kefir grain growth rate in milk was observed at an inoculation level of 1.86% [14]. In contrast, in whey-based media inoculated with 2.5%, 5.0%, and 7.5% (*w*/*v*) kefir grain concentrations, the greatest kefir grain biomass production was achieved at 5% (*w*/*v*) [17]. Furthermore, in an apple juice and whey-based fermented beverage, increasing the kefir grain concentration from 2% to 8% (*w*/*v*) led to higher acidity, and kefiran and lactic acid production, as well as elevated counts of lactobacilli and yeasts [16]. Conversely, in a whey-date beverage, the highest viability of LAB and yeasts was observed at the lowest inoculum level, within a kefir grain concentration range of 2% to 5% (*w*/*v*) [18]. Additionally, in kiwi-based kefir-like beverages, nutrient consumption, microbial growth, and the synthesis of fermentation metabolites were strongly influenced by both the shaking rate (25 to 147 rpm) and kefir grain concentration (1.32% to 4.68% *w*/*v*) [11].

Based on these observations, it is reasonable to assume that both the shaking rate and inoculum (kefir grains) concentration could also influence the aroma characteristics of fermented drinks. However, studies investigating this impact are limited.

The effects of the shaking rates and inoculum proportions on the chemical and microbiological compositions of fermented kiwifruit-based beverages were previously investigated by our research team [11]. Building on that work, the present study aimed to analyze the volatile composition of these beverages in order to provide a more comprehensive characterization. Based on the findings from both the previous and the current study, kiwifruit-based beverages exhibiting a broad aromatic profile, high probiotic cell counts, and low concentrations of sugars and ethanol were identified and selected as promising candidates for probiotic kiwi-based kefir-like products. Additionally, the microbiological and chemical stability of the selected beverages was evaluated over a 28-day refrigerated storage period at 4 °C.

## 2. Materials and Methods

### 2.1. Preparation of Inoculum and Substrate

The activation and microbial counts of the inoculum (milk kefir grains), the preparation of the kiwifruit juice, and the methods used for the microbiological and chemical characterization of the kiwifruit-based substrate and the fermented beverages Bev7-48 h and Bev3-72 h during refrigerated storage were described by Bazán et al. [11].

### 2.2. Fermentation Conditions

**Figure 1:**
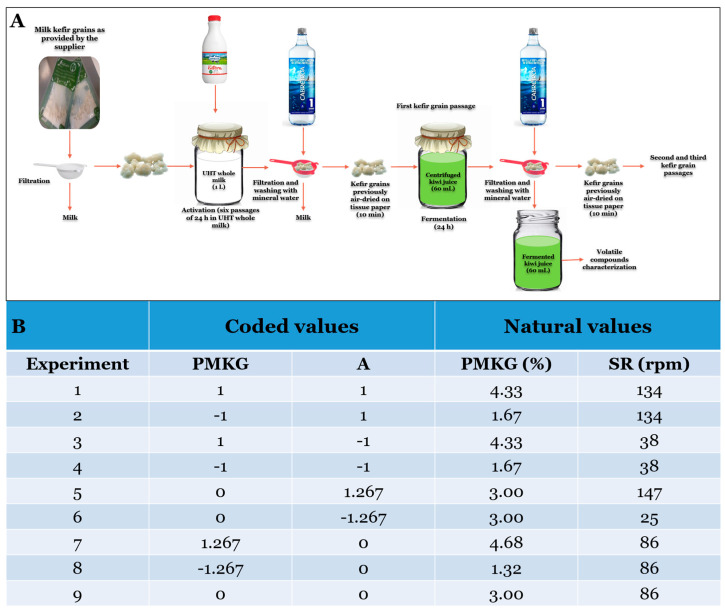
Fermentation conditions (**A**) and experimental design (**B**) for the fermentation of kiwi juice using various proportions of milk kefir grains (PMKGs) and different shaking rates (SRs) in each MKG passage. Experiments 1–8 were repeated twice, while experiment 9 was repeated five times (adapted from Bazán et al. [11]).

The fermentation conditions and experimental design are shown in Figure 1A and Figure 1B, respectively, as described by Bazán et al. [11].

### 2.3. Volatile Compounds Quantification by Gas Chromatography/Mass Spectrometry (GC/MS)

The aromatic profiles of the kiwifruit-based drinks were determined from the filtered fermented substrate (Figure 1A), following centrifugation at 5000 rpm for 5 min at 4 °C to remove precipitated material.

#### 2.3.1. Chemical Standards and Reagents

Analytical-grade reagents and chemical standards used to identify various volatile compounds were obtained from Merck (Darmstadt, Germany) and Aldrich (Buchs, Switzerland), respectively.

#### 2.3.2. Headspace Solid-Phase Microextraction

Headspace solid-phase microextraction (HS-SPME) method was used to extract volatile compounds from both unfermented and fermented kiwi juice. A 20 mL vial, containing a magnetic stirrer and 1.0 g of sodium chloride (to enhance the release of volatile organic compounds into the headspace), was filled with 5 mL of sample and 1 mL of 3-octanol (50.32 mg per 1 L of absolute ethanol). After sealing the vial with a screw cap and silicone septum, it was placed in a 60 °C water bath and shaken at 500 rpm for 2 min. This procedure was adopted because our SPME system was not equipped with a standard SPME incubation unit. Incubation using a water batch instead of the standard SPME incubation system has been widely employed in previous studies [19,20,21,22].

The fiber used for solid-phase microextraction was a 65 μm Polydimethylsiloxane/Divinylbenzene (PDMS/DVB) fiber supplied by Supelco (Bellefonte, PA, USA). This fiber had previously been successfully employed to determine the volatile composition of kiwi tissue [23], as well as the same fruit during storage and ripening [24].

This particular fiber coating was selected in the present study for several reasons: (i) it provides good reproducibility for replicated samples, and (ii) it yields a linear response (peak area compared to headspace concentration). Furthermore, the use of this fiber allowed for better recovery of volatile compounds from the studied samples [23,24].

Once conditioned at 270 °C for 30 min according to the manufacturer’s protocol, the fiber was introduced into the sample vials and kept in the headspace to retain volatile compounds for 25 min in a 60 °C water bath with agitation at 500 rpm. The adsorbed volatile compounds were then desorbed by placing the fiber into the injection port at 250 °C for 5 min in splitless mode.

#### 2.3.3. Chromatographic Conditions

The separation, identification, and quantification of volatile compounds in unfermented and fermented kiwi juice were performed using a GC 7820 A gas chromatograph (Agilent Technologies, Santa Clara, CA, USA) coupled with an Agilent MSD Series 5975 mass spectrometer. The GC-MS system was equipped with a ZB capillary column (Phenomenex; 60 m × 0.25 mm × 0.25 μm film thickness).

For the separation of volatile compounds, the initial column temperature was set to 40 °C and held for 5 min. A temperature ramp followed, increasing at 3 °C/min up to 220 °C, where it was held for 10 min. The column flow rate was maintained at 1.2 mL/min, with hydrogen used as the carrier gas. Mass spectra were scanned at 70 eV within a mass range of m/z 10 to 1000.

The volatile organic compounds (VOCs) were identified by comparing their mass spectra with those in the Wiley spectral library. When a pure volatile compound was available, its identification was further confirmed by comparing the corresponding retention times [10].

The concentrations of VOCs in the samples, expressed as means ± standard deviations from two independent experiments, each with two analytical replicates (Tables S1–S3), were determined using 3-octanol as the internal standard, based on the ratio between the peak area of each volatile compound and that of the internal standard [10].

### 2.4. Intensity of the Odor Perception of the Different VOCs

The odor intensity of various volatile organic compounds (VOCs) in unfermented and fermented kiwifruit juices was expressed as the odor activity value (OAV), defined as the ratio of the concentration of each VOC to its corresponding odor threshold reported in the literature [10]. When this ratio is equal to or greater than 1.0, the VOC is considered to significantly contribute to the aroma of the beverage. Conversely, if the OAV is less than 1.0, the compound’s influence on aroma may be either positive or negative, depending on whether it interacts synergistically or antagonistically with other VOCs in the beverage [10].

### 2.5. Statistical Analyses

#### 2.5.1. Response Surface Methodology

To produce different kefir-like beverages from kiwi juice inoculated with varying proportions of milk kefir grains (PMKGs) and incubated at different shaking rates (SRs) in each kefir grain passage, response surface methodology (RSM) was employed. The statistical significance (*p* < 0.05) of the model parameters and the overall models was evaluated using Student’s *t*-test and Fisher’s *F*-test, respectively (Tables S4–S24). Statistical analysis and the corresponding response surface plots were generated using the Statistica software package (Statistica 14.1.0 for Windows; StatSoft Inc., Tulsa, OK, USA, 2023) [11].

#### 2.5.2. Principal Component Analysis

Principal component analysis (PCA) was used to reduce the number of initial experimental variables to a smaller set of principal factors that explained most of the variance in the original data, thereby minimizing the risk of overfitting during cluster formation. The analysis was performed using IBM^®^ SPSS^®^ Statistics for Windows, Version 25.0 (IBM Corp., Armonk, NY, USA, 2017) [11]. In the first PCA, the input dataset consisted of the concentrations of 82 volatile organic compounds (VOCs). In the second PCA, the variables analyzed were the odor activity values (OAVs) of 38 VOCs. The third PCA included concentrations of total sugars, citric acid, quinic acid, lactic acid, acetic acid, ethanol, and glycerol; microbial counts of lactic acid bacteria, acetic acid bacteria, and yeasts; and the number of compounds with OAVs ≥ 1.0 (nOAVs). These principal factor values have standardized to a mean of 0 and a standard deviation of 1, ensuring that all principal components were equally weighted in the clustering process.

#### 2.5.3. Cluster Analysis

Three cluster analyses were conducted to evaluate the similarity or dissimilarity between the fresh substrate and the twenty-seven fermented beverages, using the corresponding principal factor values obtained from the three preceding PCAs.

Euclidean distance was used as the measure of similarity or dissimilarity, and the nearest neighbor linkage method (single linkage) was applied for clustering. All analyses were performed using the Cluster Analysis module in Statistica (Statistica 14.1.0 for Windows; StatSoft Inc., Tulsa, OK, USA, 2023) [11].

#### 2.5.4. Comparison of Microbiological and Chemical Variables During Refrigerated Storage

Individual experiments were performed in triplicate and the analytical determinations were performed in duplicate, with the experimental results being presented as mean ± standard deviations. Data on pH; counts of LAB, AAB, and yeasts; and concentrations of CA, QA, LA, AA, EtOH, GOH, and TS were analyzed using analysis of variance (ANOVA) with IBM SPSS Statistics for Windows, version 25.0 (IBM Corp., Armonk, NY, USA, 2017). Paired sample *t*-tests were performed to evaluate significant differences within each variable. Differences between means were considered statistically significant at a *p*-value of less than 0.05.

## 3. Results and Discussion

### 3.1. Evolution of VOC Profiles During Successive Kefir Grain Passages

As shown in Tables S1–S3, a total of 82 volatile organic compounds (VOCs) were identified across all samples, including both unfermented juice and fermented beverages. These VOCs were classified into seven chemical families: organic acids (2 compounds), alcohols (24), aldehydes (10), ketones (4), esters (24), furans (2), and a miscellaneous group labeled as “other compounds” (16).

In the unfermented kiwi juice, 14 VOCs were identified, including three alcohols, one ketone, three esters, and seven compounds classified as “other compounds”, with a total VOC concentration of 9.14 ± 0.43 mg/L (Table S1).

In the fermented samples from the first kefir grain passage, the number of VOCs was either equal to (as in Bev5-24 h) or greater than that in the unfermented juice, with the exception of Bev2-24 h, which was the only sample to exhibit a lower VOC concentration (6.12 ± 0.20 mg/L) compared to the fresh juice. Among all samples in this passage, Bev8-24 h contained the highest number of VOCs (29), while Bev6-24 h showed the highest overall concentration (20.42 ± 0.55 mg/L).

During the second kefir grain passage, only Bev5-48 h exhibited a similar number of VOCs to the unfermented juice, while all other fermented samples showed higher values. However, Bev2-48 h and Bev8-48 h had lower VOC concentrations, measuring 6.23 ± 0.21 and 8.78 ± 0.26 mg/L, respectively. The highest number of VOCs (32) was detected in both Bev3-48 h and Bev9-48 h, whereas Bev6-48 h had the highest overall concentration (21.37 ± 0.55 mg/L).

In contrast, the fermented drinks from the third batch of cultures contained both a greater number and higher concentrations of VOCs compared to the unfermented juice. Bev9-72 h exhibited the highest number of VOCs (36), and Bev6-72 h showed the highest concentration (26.94 ± 0.96 mg/L).

These results suggest that VOC production depended on the shaking rate, the inoculum size, and the number of kefir grain passages. Shaking rates ranging from 38 to 86 rpm were effective for producing fermented beverages with a high number of VOCs, as observed in Bev8-24 h (86 rpm), Bev3-48 h (38 rpm), and both Bev9-48 h and Bev9-72 h (86 rpm). The influence of the inoculum size on VOC production appeared to depend on both the shaking rate and the number of fermentation passages. For instance, the proportions of milk kefir grains for the samples with the highest VOC counts were as follows: Bev8-24 h (PMKG = 1.32% *w*/*v*), Bev3-48 h (PMKG = 4.33% *w*/*v*), and both Bev9-48 h and Bev9-72 h (PMKG = 3.00% *w*/*v*).

Moreover, the highest VOC concentrations observed in Bev6-24 h, Bev6-48 h, and Bev6-72 h suggest that a shaking rate of 25 rpm, combined with a 3.00% (*w*/*v*) inoculum, is optimal for producing kefir-like beverages rich in volatile compounds.

On the other hand, by the third passage, the kefir grains appeared to be better adapted to the acidic conditions of the unfermented kiwi juice (pH = 3.27 ± 0.17), which likely contributed to the enhanced production of volatile compounds (Tables S1–S3).

The low production of organic acids in the beverages from all three kefir grain passages, compared to other VOCs (Tables S1–S3), may be attributed to the low pH of kiwi juice, particularly in comparison to UHT whole milk (pH = 6.70; [25]). This acidic environment likely restricted the formation of organic acids. Furthermore, these compounds may have been further metabolized by yeasts present in the inoculum into other volatile compounds, such as alcohols, esters, or ketones [26].

In a previous study, kefirs were produced through 24 h batch fermentations of UHT whole milk using the same type of inoculum as employed in the present study, across four kefir grain passages [25]. The VOCs identified in both the unfermented substrate and the kefirs obtained during the first three passages included organic acids (11 compounds), alcohols (19), aldehydes (10), ketones (4), esters (22), hydrocarbons (8), and other compounds (7). The concentrations of organic acids, alcohols, aldehydes, ketones, and esters detected in the milk kefirs produced during the first three 24 h batch fermentations [25] were consistently lower than those found in the fermented kiwi-based drinks (Tables S1–S3).

A comparison of key VOCs in both types of fermented beverages during the first kefir grain passage reveals notable differences. The number of organic acids found in the fermented kiwi-based drinks (0–1; Table S1) was lower than that found in fermented UHT milk (4 compounds; [25]). The numbers of alcohols (9) and aldehydes (3) in the milk kefir fell within the ranges observed in the fermented kiwi-based beverages (alcohols: 4–11; aldehydes: 0–6). However, the number of ketones (4) and esters (14) in the milk kefir [25] exceeded those detected in the fruit-based drinks (ketones: 1–2; esters: 2–8) (Table S1).

During the second kefir grain passage, the milk kefir contained higher numbers of organic acids (6), aldehydes (6), and esters (11) [25], compared to the kiwi-based beverages, which exhibited values of 0, 0–5, and 3–12, respectively (Table S2). In contrast, the numbers of alcohols (10) and ketones (2) in the dairy beverage fell within the respective ranges observed in the fruit-based drinks (alcohols: 3–13; ketones: 1–2).

In the third kefir grain passage, the dairy beverage contained higher numbers of organic acids (10) and aldehydes (7) [25] compared to the fermented kiwi-based beverages (Table S3). However, the numbers of alcohols (10), ketones (2), and esters (13) in the milk kefir fell within the respective ranges observed in the fruit-based beverages (alcohols: 4–15, ketones: 1–2, and esters: 2–13).

### 3.2. Effect of Shaking Rate and Inoculum Percentage on the Production of VOCs in the Kiwi-Based Drinks During Successive Kefir Grain Passages

The primary objective of employing response surface methodology (RSM) in this study was to structure the experimental design for producing kefir-like beverages based on kiwifruit [11], rather than to develop empirical models describing the relationship between the production of volatile organic compounds (VOCs) and variations in the shaking rate (SR) and the proportion of milk kefir grains (PMKG). As previously mentioned, a total of 82 VOCs were identified across the 27 fermented beverage samples, making it both impractical and labor-intensive to evaluate the effects of the two independent variables on the concentrations of each individual compound.

Furthermore, the application of RSM to evaluate the effects of the SR and PMKG on specific VOC families—such as total organic acids, alcohols, aldehydes, ketones, esters, furans, and others—resulted in empirical models that were not consistently statistically significant (see Tables S4–S21). In fact, only the total concentration of volatile compounds ([TVOCs]) in each beverage, measured across successive kefir grain passages, could be reliably modeled as a function of the two independent variables.

The resulting empirical models for [TVOCs] obtained at 24, 48, and 72 h were as follows:

[TVOCs] (24 h) = 13.92 − 3.25·SR + 2.04·PMKG − 2.59·PMKG^2^ (1)

[TVOCs] (48 h) = 13.86 − 1.61·SR + 1.40·PMKG − 3.25·PMKG^2^ (2)

[TVOCs] (72 h) = 24.60 − 1.93·SR + 1.48·PMKG − 4.87·PMKG^2^ (3)

The three empirical models and their parameter values demonstrated a consistently good fit, as confirmed by both Student’s *t*-test and Fisher’s *F*-test (see Tables S22–S24 in the Supplementary Materials).

**Figure 2:**
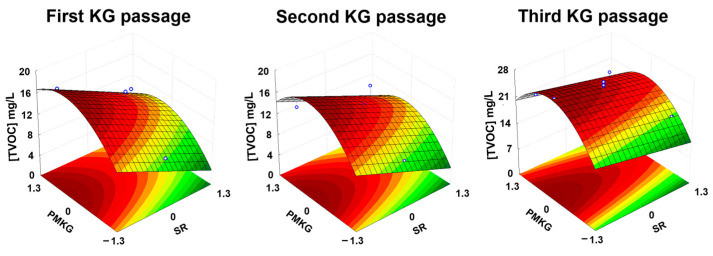
Response surface plots generated from empirical models 1, 2, and 3, illustrating the effects of shaking rate (SR) and the proportion of milk kefir grains (PMKG) on the total concentration of volatile compounds ([TVOCs]) in 27 kefir-like beverages during the first, second, and third kefir grain (KG) passages.

A general trend observed across models 1–3 is that the coefficients for the linear terms of SR and PMKG consistently exhibited negative and positive signs, respectively. In contrast, the coefficients for the quadratic term of the SR and for the interaction between the SR and PMKG were not statistically significant in any of the models. The coefficient for the quadratic term of the PMKG, however, was negative in all cases. Consequently, the optimal value for the SR was consistently at its minimum (25 rpm), while the optimal values for the PMKG fell within the tested experimental range (Tables S22–S24): 3.52%, 3.29%, and 3.20% for the first, second, and third kefir grain passages, respectively (Figure 2). At these optimal conditions, the maximum total volatile organic compound (TVOC) concentrations predicted by the models were 18.55, 17.70, and 27.22 mg/L for the first, second, and third passages, respectively.

While shaking rates above 25 rpm may enhance mass transfer into the substrate, they can also promote the loss of volatile compounds from the fermented beverage to the surrounding air, thereby reducing their concentration in the final product. Moreover, excessive agitation may damage the kefir grains, negatively impacting their growth and metabolite production [11]. The observed increase in total VOC concentration with rising PMKG levels—up to the optimal point—could be attributed to a greater number of microbial cells introduced into the substrate [11]. In contrast, the decline in VOC concentrations beyond the optimal PMKG level may result from the increased competition among microbial cells for nutrients or from the production of inhibitory compounds [11], both of which can limit microbial growth and VOC synthesis.

Given the complex microbiota of kefir grains—comprising various species from three major microbial groups (lactic acid bacteria [LAB], acetic acid bacteria [AAB], and yeasts)—and the intricate interactions among them, it is difficult to attribute the production of a specific volatile compound to a particular species or microbial group. Consequently, the effects of the two independent variables (SR and PMKG) on the total concentration of volatile compounds cannot be directly linked to their influence on the growth of LAB, AAB, or yeasts. For instance, Bazan et al. [11] reported that increasing the shaking rate above 25 rpm promoted the growth of AAB and yeasts and enhanced the total sugar consumption, while inhibiting LAB growth and differentially affecting the production of organic acids and alcohols.

The large number of volatile organic compounds (VOCs) detected (82 in total) in the unfermented kiwifruit juice (UKJ) and the 27 fermented beverages produced across three kefir grain passages (Tables S1–S3) made it difficult to detect the similarities and differences among these drinks. For this reason, a principal component analysis (PCA) was performed to reduce the dimensionality of the dataset. The analysis identified 21 principal components with eigenvalues greater than 1.0, collectively explaining 95.73% of the total variance (Table S25). The 21 principal factor values obtained from this analysis were then used in a cluster analysis to identify the similarities and differences among the samples.

**Figure 3:**
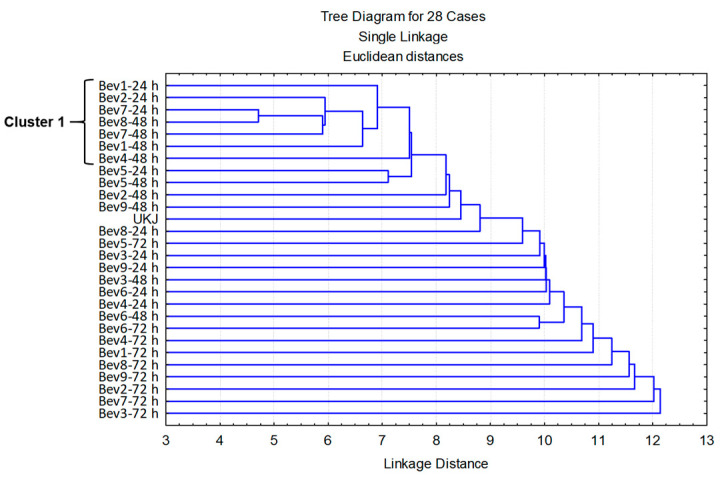
Clusters formed by the unfermented kiwifruit juice (UKJ) and the 27 beverages obtained in the first, second, and third kefir grain passages (Tables S1–S3), based on the 21 factors obtained from the first PCA.

As shown in Figure 3, a distinct subcluster of seven beverages emerged from the analysis of the 21 principal factor values used as classification variables. This subcluster initially grouped Bev7-24 h (SR = 86 rpm, PMKG = 4.68%, *w*/*v*) and Bev8-48 h (SR = 86 rpm, PMKG = 1.32%, *w*/*v*) at the smallest distance, indicating that these two beverages had the most similar volatile profiles. Notably, the kefir-like beverages Bev7-24 h and Bev8-48 h contained 25 (Table S1) and 24 (Table S2) volatile compounds, respectively, but at relatively low concentrations.

The remaining beverages—Bev7-48 h (SR = 86 rpm, PMKG = 4.68%, *w*/*v*), Bev2-24 h (SR = 134 rpm, PMKG = 1.67%, *w*/*v*), Bev1-48 h and Bev1-24 h, both obtained at SR = 134 rpm and PMKG = 4.33%, *w*/*v*, and Bev4-48 h (SR = 38 rpm, PMKG = 1.67%, *w*/*v*)—were subsequently added to this subcluster in order of increasing distance indices.

Next, an independent cluster formed by Bev5-24 h and Bev5-48 h—both produced at SR = 147 rpm and PMKG = 3.00% *w*/*v*—was incorporated into cluster 1, although it exhibited a higher distance index than the initial grouping. The remaining beverages were subsequently added to cluster 1 in order of increasing distance index.

The most distinct beverages were eight samples from the third kefir grain passage (Bev3-72 h, Bev7-72 h, Bev2-72 h, Bev9-72 h, Bev8-72 h, Bev1-72 h, and Bev4-72 h), which showed the greatest differences in volatile compound concentrations compared to the other drinks. This distinction is primarily attributed to their higher number of volatile compounds and/or the presence of these compounds at higher concentrations (Tables S1–S3).

However, the volatile composition of each sample includes compounds that may or may not contribute to the aromatic properties of the kiwifruit-based beverages [10,21]. With this in mind, the next analysis focused on exploring the relationships among the 28 beverages using the odor activity values (OAVs) of 38 volatile compounds with known odor descriptors and thresholds (Table 1) as classification variables. This approach enables the identification of similarities in the potential aroma profiles of the kiwi beverages.

Prior to the cluster analysis, a principal component analysis (PCA) was performed using the OAVs of the 38 VOCs with OAVs ≥ 1.0. This analysis yielded 12 principal components with eigenvalues greater than 1.0, collectively explaining 88.51% of the total variance (Table S26). As with the previous PCA, the 12 factor values from this analysis were used in a cluster analysis to group the 28 beverages.

**Figure 4:**
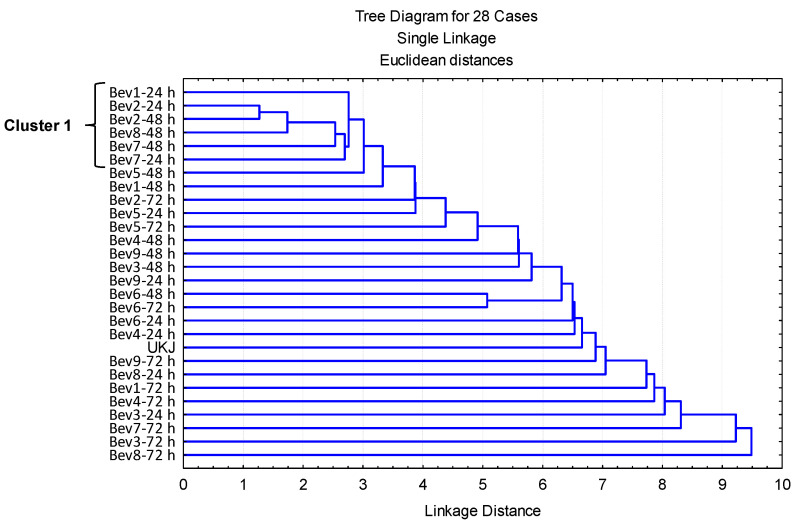
Clusters formed by the substrate (UKJ) and the fermented fruit-based beverages, based on the 12 factors obtained from the second PCA.

As shown in Figure 4, a single cluster comprising six beverages was identified. Within this cluster, three beverages—Bev2-24 h, Bev2-48 h, and Bev8-48 h—were initially grouped together based on the smallest distance index. Subsequently, Bev7-48 h, Bev7-24 h, and Bev1-24 h were added to this subcluster. These beverages contained six (Bev2-24 h), seven (Bev1-24 h), and eight (Bev2-48 h, Bev8-48 h, Bev7-48 h, and Bev7-24 h) volatile compounds with OAVs greater than 1.0 (Table 2, Table 3 and Table 4).

The volatile compounds with OAVs > 1.0 identified in these six beverages included 1-pentanol (present in all six beverages), 1-hexanol (Bev7-24 h and Bev2-48 h), 2-phenylethanol (Bev7-24 h Bev2-48 h, and Bev8-48 h), 2-undecanol (Bev7-48 h and Bev8-48 h), (E)-2-nonenal (Bev7-24 h and Bev8-48 h), and 4-hydroxy-3-methoxybenzaldehyde (vanillin) (all six). Other compounds included 2,6-dimethyl-4-heptanone (all six), pentyl acetate (Bev7-24 h), methyl benzoate (Bev1-24 h, Bev7-24 h, Bev2-48 h, and Bev8-48 h), ethyl octanoate (Bev1-24 h, Bev2-24 h, Bev2-48 h, and Bev7-48 h), ethyl decanoate (Bev7-48 h), ethyl hexanoate (Bev1-24 h and Bev7-48 h), and 2,5-dimethyl-4-hydroxy-3(2H)-furanone (all six). As a result, these beverages are characterized by fruity, sweet, floral, rose-like, creamy vanilla-like, and caramel-like aromas. However, additional unpleasant resinous notes were detected in Bev7-24 h and Bev2-48 h, while fatty notes were present in Bev7-24 h and Bev8-48 h. Furthermore, all six beverages exhibited pungent, fermented, bready, yeasty, fusel, oily, winey, and solvent-like odor descriptors.

In contrast, Bev8-72 h, Bev3-72 h, Bev7-72 h, Bev3-24 h, Bev4-72 h, Bev1-72 h, Bev8-24 h, and Bev9-72 h were the most distinct beverages (Figure 4), each containing 7, 14, 13, 10, 14, 11, 7, and 17 volatile compounds, respectively, with OAVs considerably greater than 1.0 (Table 2, Table 3 and Table 4).

The volatile compounds detected in these beverages included 1-pentanol (present in all eight beverages), 2-methyl-1-propanol (Bev1-72 h and Bev9-72 h), 3-methyl-1-pentanol (Bev3-24 h and Bev7-72 h), 1-dodecanol (Bev3-24 h, Bev3-72 h, Bev4-72 h, and Bev9-72 h), 1-hexanol (Bev4-72 h and Bev9-72 h), 1-octanol (Bev9-72 h), 2-phenylethanol (Bev1-72 h, Bev3-72 h, Bev4-72 h, Bev7-72 h, Bev8-72 h, and Bev9-72 h), 2-heptanol (Bev9-72 h), 2-undecanol (Bev8-24 h, Bev3-72 h, Bev4-72 h, Bev7-72 h, Bev8-72 h, and Bev9-72 h), (E)-2-hexenal (Bev4-72 h and Bev9-72 h), (E)-2-nonenal (Bev3-72 h, Bev4-72 h, Bev7-72 h, Bev8-72 h, and Bev9-72 h), 2-methylbutanal (Bev8-24 h and Bev8-72 h), 4-hydroxy-3-methoxybenzaldehyde (Bev3-24 h, Bev8-24 h, Bev1-72 h, Bev3-72 h, Bev4-72 h, Bev7-72 h, and Bev9-72 h), 2,6-dimethyl-4-heptanone (present in all eight beverages), 2-heptanone (Bev3-24 h), 4-methyl-2-hexanone (Bev8-24 h), 2-methylbutyl acetate (Bev1-72 h and Bev7-72 h), pentyl acetate (Bev3-72 h), ethyl 3-phenylpropanoate (Bev1-72 h), ethyl butanoate (Bev9-72 h), methyl benzoate (Bev3-24 h, Bev1-72 h, Bev3-72 h, Bev7-72 h, and Bev9-72 h), ethyl decanoate (Bev3-72 h, Bev4-72 h, Bev7-72 h, and Bev9-72 h), ethyl hexanoate (Bev3-24 h, Bev1-72 h, Bev3-72 h, Bev4-72 h, and Bev7-72 h), ethyl octanoate (Bev3-24 h, Bev1-72 h, Bev3-72 h, Bev4-72 h, Bev7-72 h, and Bev9-72 h), and 2,5-dimethyl-4-hydroxy-3(2H)-furanone (present in all eight beverages).

Thus, the broader aromatic profile of these beverages includes fruity, sweet, and caramel-like notes. In addition, several pleasant aromas were identified, such as malt (Bev1-72 h and Bev9-72 h), wine-like (Bev3-24 h and Bev7-72 h), raw carrot (Bev3-24 h, Bev3-72 h, Bev4-72 h, and Bev9-72 h), soapy (Bev9-72 h), honey (Bev1-72 h, Bev3-72 h, Bev4-72 h, Bev7-72 h, Bev8-72 h, and Bev9-72 h), nutty (Bev8-24 h and Bev8-72 h), and creamy vanilla-like (Bev3-24 h, Bev8-24 h, Bev1-72 h, Bev3-72 h, Bev4-72 h, Bev7-72 h, and Bev9-72 h). However, pungent, fermented, bready, yeasty, fusel, oily, winey, and solvent-like odors were also present across all eight beverages. Some samples exhibited unpleasant notes, including resinous (Bev4-72 h and Bev9-72 h), bitter almond-like (Bev4-72 h and Bev9-72 h), and fatty odors (Bev3-72 h, Bev4-72 h, Bev7-72 h, Bev8-72 h, and Bev9-72 h).

Additionally, the beverages Bev5-24 h and Bev5-48 h exhibited the poorest aromatic profiles, with only four volatile compounds having an OAV greater than 1.0.

These findings confirm that variations in the shaking rate and inoculum proportion can significantly influence the volatile composition, resulting in kefir-like beverages with unique aromatic profiles and distinct sensory characteristics.

### 3.3. Relationships Among the Microbiological, Chemical, and Volatile Odorant Compositions of Beverages from the Three Kefir Grain Passages

**Figure 5:**
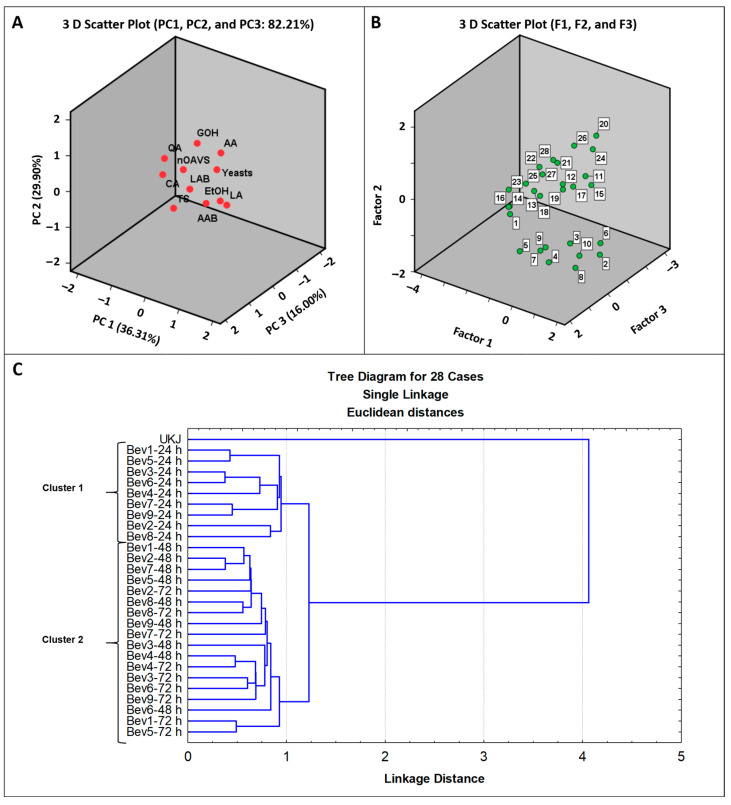
(**A**): Projection of the independent variables (TS: total sugars, CA: citric acid, QA: quinic acid, LA: lactic acid, AA: acetic acid, EtOH: ethanol, GOH: glycerol, LAB: lactic acid bacteria count, AAB: acetic acid bacteria count), yeasts count, and the number of VOCs with OAVs ≥ 1.0 [nOAVs] from Table 2, Table 3 and Table 4 on a 3D scatterplot, as a function of PC1, PC2, and PC3. (**B**): Projection of the 28 kiwifruit-based beverages on the 3D scatterplot, as a function of Factor 1, Factor 2, and Factor 3. Beverages 2–10, 11–19, and 20–28 correspond to those obtained during the first, second, and third kefir grain passages, respectively, following the experimental order outlined in Figure 1B. Beverage 1 is the unfermented kiwi juice (UKJ). (**C**): Clusters formed by the unfermented kiwi juice and the 27 fermented kiwifruit-based beverages, using the values of Factors 1, 2, and 3 from the PCA as classification variables.

To complete the present study, a third PCA was conducted based on the microbiological and chemical compositions [11] and the number of volatile compounds with OAVs greater than 1.0 (Table 2, Table 3 and Table 4) in the 28 kiwifruit-based beverages. This analysis included the counts of LAB, AAB, and yeasts, as well as the concentrations of citric acid, quinic acid, lactic acid, acetic acid, ethanol, glycerol, and total sugars [11]. Using this approach, the initial 11 independent variables were reduced to three principal components (PC1, PC2, and PC3) with eigenvalues greater than 1.0 (Table S27). These components accounted for 36.31%, 29.90%, and 16.00% of the variance, respectively. Together, the three components explained 82.21% of the total variance (Figure 5A, Table S27).

The first principal component (PC1) was positively correlated, in descending order, with the AAB count, lactic acid concentration, and yeast count, and negatively correlated with the citric and quinic acid levels. The second principal component (PC2) showed positive correlations with the glycerol, acetic acid, and yeast count, and negative correlations with the total sugar and lactic acid concentrations. The third principal component (PC3) was primarily characterized by a high LAB count and a greater number of volatile compounds with odor activity values (OAVs) above 1.0. Figure 5B shows the distribution of the 28 beverages based on their scores for the three factors obtained from the PCA.

Given the complexity of interpreting the grouping of the 28 kiwifruit-based beverages based on PCA (Figure 5A,B), a third cluster analysis was performed using the three principal factors to prevent overfitting in the formation of clusters.

As shown in Figure 5C, two distinct clusters were observed. The first cluster comprised nine beverages from the first kefir grain passage, while the second cluster included the eighteen beverages from the second and third kefir grain passages. As expected, the unfermented kiwi juice was notably distinct, standing out as the most different beverage (Figure 5C).

In the first cluster, the number of volatile compounds with an OAV greater than 1.0 ranged from four to twelve: Bev5-24 h (four compounds), Bev2-24 h (six compounds), Bev1-24 h and Bev8-24 h (seven compounds), Bev7-24 h (eight compounds), Bev6-24 h and Bev9-24 h (nine compounds), Bev3-24 h (ten compounds), and Bev4-24 h (twelve compounds).

In the second cluster, Bev5-48 h (four compounds) and Bev5-72 h (five compounds) contained the fewest volatile compounds with OAV > 1.0, while Bev6-48 h, Bev3-72 h, and Bev4-72 h, each with fourteen compounds, as well as Bev9-72 h (seventeen compounds), had the highest number of aromatic compounds (Table 2, Table 3 and Table 4).

Interestingly, the nine beverages from the first kefir grain passage exhibited higher concentrations of total sugars (71.10–107.53 g/L), lactic acid (0.41–1.31 g/L), ethanol (0.36–4.50 g/L), and AAB counts (5.88–6.93 log colony-forming units (CFU)/mL), while they had lower levels of quinic acid (5.49–7.92 g/L), acetic acid (0.07–0.22 g/L), and glycerol (0.18–0.21 g/L) compared to the beverages in the second cluster. All beverages in the second cluster had yeast counts exceeding 10^6^ CFU/mL [11], which is the threshold considered to confer probiotic benefits on the host [63].

A detailed analysis of the experimental data revealed that the beverages Bev4-24 h and Bev6-24 h, which contained twelve and nine aromatic compounds with an OAV > 1.0, respectively, had the highest total sugar concentrations (107.53 and 102.66 g/L, respectively). These values represented 87.02% and 83.09% of the initial total sugar concentration in the unfermented kiwi juice. This suggests that Bev4-24 h and Bev6-24 h are high-calorie beverages, and their consumption could contribute to excessive calorie intake. Notably, while Bev6-24 h contained counts of LAB, AAB, and yeasts exceeding 10^6^ CFU/mL, Bev4-24 h had an LAB count higher than 10^6^ CFU/mL, with AAB and yeast counts slightly below this threshold.

Conversely, Bev7-72 h and Bev1-72 h, which contain thirteen and eleven volatile compounds with an OAV greater than 1.0 (Table 4), had the lowest calorie content due to their reduced TS concentrations (31.12 and 34.13 g/L, respectively). This indicates that 74.81% and 72.38% of the initial sugars in the substrate were consumed during fermentation. However, in both beverages, only the yeast count exceeded 10^6^ CFU/mL [11].

Although two beverages (Bev5-24 h and Bev9-24 h) contained counts of all three microbial groups exceeding 10^6^ CFU/mL, their sugar content was above 71 g/L. In contrast, nine beverages (Bev8-24 h, Bev3-48 h, Bev4-48 h, Bev6-48 h, Bev7-48 h, Bev8-48 h, Bev9-48 h, Bev3-72 h, and Bev6-72 h) had LAB and yeast counts, which are associated with recognized probiotic effects [7,8], exceeding 10^6^ CFU/mL. Among these, Bev7-48 h and Bev3-72 h had the lowest sugar concentrations (<57 g/L). Notably, Bev3-72 h exhibited a broader aromatic profile, with 14 volatile compounds having OAV > 1.0, compared to Bev7-48 h, which contained 8 volatile compounds with OAV > 1.0 (Table 3 and Table 4). Both beverages were subsequently identified as suitable functional foods.

### 3.4. Microbiological and Chemical Analysis of Beverages Bev7-48 h and Bev3-72 h During 7, 14, 21, and 28 Days of Refrigerated Storage

After selecting the most suitable beverages based on their chemical, microbiological, and aromatic characteristics, the subsequent study focused on evaluating their stability under storage conditions.

Table 5 illustrates the changes in the microbiological and chemical compositions of the two selected beverages during refrigerated storage at 4 °C over 7, 14, 21, and 28 days. As shown, the pH values of beverage Bev7-48 h remained largely unchanged (*p* > 0.05) during refrigerated storage, while only minor changes (*p* < 0.05) were observed in Bev3-72 h.

LAB counts remained relatively stable in both beverages, consistently staying above 10^6^ CFU/mL throughout the storage period. The final counts were 2.1 × 10^6^ CFU/mL for Bev7-48 h and 1.4 × 10^7^ CFU/mL for Bev3-72 h. In contrast, AAB counts exhibited a declining trend, particularly after day 7 in Bev7-48 h and progressively throughout the entire storage period in Bev3-72 h (*p* < 0.05). Yeast counts, on the other hand, showed a slight increase (*p* < 0.05), with growth rates of 0.015 and 0.006 log CFU/mL/day, reaching final levels of 5.2 × 10^6^ and 1.6 × 10^7^ CFU/mL, respectively, by the end of the storage period (Table 5).

These findings suggest that storage at 4 °C, coupled with the acidic pH of the beverages, did not significantly affect LAB viability, but it gradually reduced the viability of AAB cells and promoted yeast growth.

Regarding organic acids, both Bev7-48 h and Bev3-72 h exhibited a slight but significant decrease (*p* < 0.05) in the concentrations of citric acid (0.11 and 0.10 g/L/day, respectively) and quinic acid (0.05 g/L/day in both cases). In contrast, the levels of lactic and acetic acids increased slightly (*p* < 0.05) from day 0 to day 28. Lactic acid rose at rates of 0.005 g/L/day in Bev7-48 h and 0.010 g/L/day in Bev3-72 h, while acetic acid increased at a rate of 0.010 g/L/day in both beverages.

The concentrations of alcohols increased significantly (*p* < 0.05) during storage, with ethanol levels rising at rates of 0.017 g/L/day in Bev7-48 h and 0.008 g/L/day in Bev3-72 h, while glycerol levels increased at rates of 0.011 g/L/day and 0.007 g/L/day, respectively. This increase contrasts with the significant decrease (*p* < 0.05) in total sugar concentrations in both beverages (Table 5), at rates of 0.29 g/L/day for Bev7-48 h and 0.35 g/L/day for Bev3-72 h.

These results suggest that, during storage at 4 °C for 28 days, the microorganisms in the fermented beverages continued to metabolize nutrients and produce metabolic by-products, albeit at much slower rates than those observed during the active fermentation phase.

Comparable findings have been reported in previous studies. Buran et al. [64] observed reductions in pH values and viable cell counts of *Lactobacillus acidophilus* and *Bifidobacterium bifidum* in kefir samples made from cow and goat milk after 28 days of refrigerated storage at 4 °C. In contrast, another study [65] reported no significant changes in pH, total sugar concentrations, or counts of yeasts and AAB during the storage of milk kefir at 5 ± 1 °C over 28 days. However, LAB counts declined between days 7 and 14 before stabilizing above 6.0 log CFU/mL.

Similarly, Bazán et al. [25] noted slight decreases in pH, lactose content, and the viability of the three microbial groups in milk kefir stored at 4 °C for 28 days, while the concentrations of lactic acid, acetic acid, ethanol, and glycerol increased over the same period.

Taken together, the findings from this study and previous research [25,64,65] suggest that the evolution of fermented beverages during refrigerated storage is influenced by their microbiological and chemical compositions.

## 4. Conclusions

The results of this study demonstrate the feasibility of producing potentially probiotic, low-calorie, and low-alcohol kiwi-based beverages with a broader aromatic profile, making them suitable for human consumption. This approach offers several advantages. First, it expands the range of kefir-like beverages that can be produced by fermenting fruit juices with milk kefir grains, traditionally used for milk kefir production. Second, it facilitates the incorporation of kiwifruit into a simple, cost-effective production process, potentially enabling both the homemade and industrial-scale production of a high-value fermented beverage. Third, the commercialization and harvesting of kiwifruit could stimulate agricultural activity, increasing farmers’ income and creating new job opportunities.

Moreover, the evolution of pH, nutrients, and metabolic by-products in the two selected beverages during refrigerated storage suggests that proper preservation is achieved, which supports efficient distribution from production centers to retail outlets.

To complete this study, the sensory evaluation of the selected beverages, along with other fruit-based kefir-like drinks, and the assessment of their volatile profile stabilities will be conducted in our laboratory.

## Figures and Tables

**Table 1:** Odor descriptors and odor detection thresholds (ODTs in mg/L) of volatile organic compounds detected in nonfermented kiwifruit juice (RTGJ) and various kiwi kefir-like beverages, as reported in the literature.

No.	Compound	Odor Descriptor	ODT
	Organic acids		
2	Octanoic acid	Sweat, cheese [27]	0.50 [28]
	Alcohols		
3	1-Dodecanol	Raw carrot [29]	0.016 [30]
6	1-Hexadecanol	Floral, waxy [31]	0.75 [32]
7	1-Hexanol	Resin, flower, green [31,33]	0.0056 [34]
8	1-Octanol	Soap, fruity [29,35]	0.1258 [36]
9	1-Pentanol	Pungent, fermented, bready, yeasty, fusel, oil, winey, solvent [37]	0.12 [38]
10	1-Undecanol	Fruity [35]	0.70 [39]
15	2-Heptanol	Mushroom-like [36], green [40]	0.06523 [34]
16	2-Hexanol	Fatty, fruity [36]	1.5082 [36]
17	2-Methyl-1-propanol	Malt [41]	0.55 [34]
18	2-Nonanol	Green, fruity [29,42]	0.07 [39]
19	2-Phenylethanol	Honey [36], floral, rose-like [43]	0.5642 [34]
20	2-Undecanol	Fruity [42]	0.041 [42]
21	3-Methyl-1-pentanol	Wine, green [36]	0.0075 [34]
26	Furfuryl alcohol	Sugar burnt [44]	4.50 [34]
	Aldehydes		
27	(E)-2-Hexenal	Floral, grass, green apple-like, bitter almond-like [41,45]	0.11 [34]
28	(E)-2-Nonenal	Fatty, green [41]	0.000295 [34]
32	2-Methylbutanal	Nutty [36]	0.001 [34]
33	4-Hydroxy-3-methoxybenzaldehyde	Sweet [41], creamy vanilla-like [46]	0.053 [41]
34	5-Hydroxymethylfurfural	Sweet, caramel [47]	1.00 [34]
35	Benzaldehyde	Bitter almond [29,36]	0.75089 [36]
36	Furfural	Wood, almond [36]	14.10 [28]
	Ketones		
37	2,6-Dimethyl-4-heptanone	Fruity, sweet [48]	0.11 [49]
38	2-Heptanone	Banana, fruity, floral and musty, fresh cream flavor [29]	0.005 [50]
39	4-Methyl-2-hexanone	Fruity [51]	0.002455 [34]
	Esters		
43	2-Methylbutyl acetate	Banana, fruity [52]	0.16 [52]
44	2-Phenylethyl acetate	Flowery [53], fruity, cooked apple, marmalade [54]	0.24959 [34]
52	Ethyl 3-phenylpropanoate	Fruity, floral [55]	0.014 [46]
53	Ethyl butanoate	Fruity, apple-like, banana-like, sweet, fragrant [36,56]	0.0009 [36]
54	Ethyl decanoate	Fruity [56]	0.2 [28]
55	Ethyl dodecanoate	Fruity, floral [56]	5.9 [34]
56	Ethyl hexadecanoate	Fruity, creamy, waxy [57]	2.00 [58]
57	Ethyl hexanoate	Fruity, pineapple-like [59]	0.014 [28]
58	Ethyl octanoate	Sweet [35], fruity, floral [43]	0.005 [43]
59	Hexyl acetate	Banana [29], green apple, sweet [60]	0.67 [52]
60	Methyl benzoate	Fruity, sweet [53]	0.00052 [53]
64	Pentyl acetate	Fruity [56]	0.043 [56]
	Furans		
65	2,5-Dimethyl-4-hydroxy-3(2H)-furanone	Caramel-like [61]	0.01 [62]

**Table 2:** Odor activity values (OAVs) of the volatile organic compounds detected in the unfermented kiwifruit juice (UKJ) and in different kiwi kefir-like beverages obtained from the first kefir grain passage (0–24 h).

No.	Compound	UKJ	Bev1-24 h	Bev2-24 h	Bev3-24 h	Bev4-24 h	Bev5-24 h	Bev6-24 h	Bev7-24 h	Bev8-24 h	Bev9-24 h
	Organic acids										
1	Octanoic acid	–	0.66	–	–	–	–	–	–	–	–
	Alcohols										
2	1-Dodecanol	–	–	–	18.75	35.63	–	–	–	–	–
3	1-Hexadecanol	–	0.56	–	–	0.48	–	–	0.17	0.17	1.04
4	1-Hexanol	33.93	–	–	–	–	28.57	–	23.21	–	–
5	1-Octanol	–	–	–	–	–	–	–	–	–	1.51
6	1-Pentanol	–	10.42	5.17	19.00	1.08	4.08	17.83	19.67	1.50	5.92
7	1-Undecanol	–	0.50	0.07	0.27	0.31	–	0.71	0.44	0.89	0.20
8	2-Heptanol	–	–	–	–	–	–	–	–	–	–
9	2-Hexanol	–	–	–	–	–	–	–	–	–	–
10	2-Methyl-1-propanol	–	–	–	–	–	–	2.07	0.33	–	–
11	2-Nonanol	–	–	–	–	1.86	–	–	–	–	–
12	2-Phenylethanol	0.28	0.85	1.49	0.28	0.04	0.25	0.73	0.62	0.21	2.02
13	2-Undecanol	–	–	–	–	1.22	–	6.59	–	5.85	12.68
14	3-Methyl-1-pentanol	–	–	–	22.67	–	–	–	–	–	–
15	Furfuryl alcohol	0.22	0.03	0.02	–	0.03	0.17	–	0.03	0.15	–
	Aldehydes										
16	(E)-2-Hexenal	–	–	–	–	–	–	–	–	–	–
17	(E)-2-Nonenal	–	–	–	–	203.39	–	1694.92	237.29	–	745.76
18	2-Methylbutanal	–	–	–	–	–	–	–	–	40.00	–
19	4-Hydroxy-3-methoxybenzaldehyde	–	4.72	4.15	8.49	4.15	–	14.53	5.28	3.02	6.60
20	5-Hydroxymethylfurfural	–	–	–	–	–	–	–	–	0.46	–
21	Benzaldehyde	–	0.33	0.25	0.67	0.37	–	1.08	0.53	0.20	0.83
22	Furfural	–	–	–	–	–	–	–	–	0.01	–
	Ketones										
23	2,6-Dimethyl-4-heptanone	32.82	9.64	5.64	11.09	7.82	9.27	16.27	8.55	8.82	17.18
24	2-Heptanone	–	–	–	50.00	20.00	–	–	–	–	–
25	4-Methyl-2-hexanone	–	–	–	–	–	–	–	–	69.25	–
	Esters										
26	2-Methylbutyl acetate	–	–	–	–	–	–	–	–	–	–
27	2-Phenylethyl acetate	–	0.36	–	–	–	–	–	0.32	–	–
28	Ethyl 3-phenylpropanoate	–	–	–	–	–	–	–	–	–	–
29	Ethyl butanoate	788.89	–	–	–	–	–	–	–	–	–
30	Ethyl decanoate	–	–	–	–	0.10	–	–	0.50	–	–
31	Ethyl dodecanoate	–	0.01	–	–	–	–	–	–	–	0.04
32	Ethyl hexadecanoate	–	–	–	–	–	–	–	–	–	–
33	Ethyl hexanoate	42.86	25.71	–	23.57	9.29	47.14	–	–	–	–
34	Ethyl octanoate	–	64.00	38.00	54.00	176.00	–	120.00	–	–	–
35	Hexyl acetate	–	–	–	–	–	–	–	–	–	–
36	Methyl benzoate	–	269.23	–	692.31	307.69	–	–	423.08	–	–
37	Pentyl acetate	–	–	–	–	–	–	–	2.56	–	–
	Furans										
38	2,5-Dimethyl-4-hydroxy-3(2H)-furanone	–	14.00	20.00	203.00	27.00	–	51.00	22.00	10.00	110.00
	**Number of VOCs with OAVs ≥ 1.0**	4	7	6	10	12	4	9	8	7	9

**Table 3:** Odor activity values (OAVs) of the volatile organic compounds detected in the different kiwi kefir-like beverages obtained from the second kefir grain passage (24–48 h).

No.	Compound	Bev1-48 h	Bev2-48 h	Bev3-48 h	Bev4-48 h	Bev5-48 h	Bev6-48 h	Bev7-48 h	Bev8-48 h	Bev9-48 h
	Organic acids									
1	Octanoic acid	–	–	–	–	–	–	–	–	–
	Alcohols									
2	1-Dodecanol	6.25	–	38.75	19.38	–	–	–	–	6.25
3	1-Hexadecanol	0.57	–	0.45	0.35	–	–	–	0.41	0.68
4	1-Hexanol	–	5.36	–	26.79	–	–	–	–	–
5	1-Octanol	–	–	–	–	–	–	–	–	–
6	1-Pentanol	26.25	11.92	16.58	2.92	12.00	31.75	25.75	13.58	38.00
7	1-Undecanol	0.27	0.14	0.19	–	–	1.74	0.13	0.34	0.40
8	2-Heptanol	–	–	–	–	–	–	–	–	2.61
9	2-Hexanol	–	–	–	–	–	–	–	–	–
10	2-Methyl-1-propanol	0.58	0.36	0.53	–	–	1.91	0.24	0.56	0.49
11	2-Nonanol	–	–	–	–	–	–	–	–	–
12	2-Phenylethanol	1.35	1.08	0.76	0.11	1.38	2.23	0.92	1.06	1.91
13	2-Undecanol	1.95	–	–	6.59	–	6.83	1.71	2.44	4.63
14	3-Methyl-1-pentanol	–	–	–	–	–	–	–	–	–
15	Furfuryl alcohol	0.04	–	–	0.03	–	0.04	0.03	–	–
	Aldehydes									
16	(E)-2-Hexenal	–	–	–	–	–	–	–	–	1.00
17	(E)-2-Nonenal	–	–	1254.24	271.19	–	1457.63	–	338.98	372.88
18	2-Methylbutanal	–	–	–	–	60.00	–	–	–	–
19	4-Hydroxy-3-methoxybenzaldehyde	2.45	3.77	11.51	5.47	–	6.23	3.96	4.72	5.28
20	5-Hydroxymethylfurfural	–	–	–	–	–	–	–	–	–
21	Benzaldehyde	0.17	0.21	0.77	0.39	–	0.71	0.29	0.31	0.41
22	Furfural	–	–	–	–	–	–	–	–	–
	Ketones									
23	2,6-Dimethyl-4-heptanone	5.82	4.18	3.73	9.55	12.00	10.73	4.27	6.09	7.55
24	2-Heptanone	–	–	–	42.00	–	–	–	–	–
25	4-Methyl-2-hexanone	–	–	–	–	–	–	–	–	–
	Esters									
26	2-Methylbutyl acetate	1.38	–	0.94	–	–	–	–	–	–
27	2-Phenylethyl acetate	0.12	–	–	–	0.68	1.20	0.20	–	–
28	Ethyl 3-phenylpropanoate	–	–	–	–	–	–	–	–	–
29	Ethyl butanoate	–	–	–	–	–	–	–	–	–
30	Ethyl decanoate	–	–	1.35	–	–	6.10	1.15	–	0.90
31	Ethyl dodecanoate	–	–	0.02	–	–	0.10	0.02	–	0.03
32	Ethyl hexadecanoate	–	–	–	–	–	–	–	–	–
33	Ethyl hexanoate	73.57	–	29.29	9.29	–	42.14	8.57	–	–
34	Ethyl octanoate	24.00	22.00	56.00	98.00	–	86.00	92.00	–	52.00
35	Hexyl acetate	–	–	–	–	–	–	–	–	–
36	Methyl benzoate	365.38	134.62	288.46	–	–	–	–	211.54	–
37	Pentyl acetate	1.40	–	–	–	–	8.37	–	–	–
	Furans									
38	2,5-Dimethyl-4-hydroxy-3(2H)-furanone	10.00	16.00	58.00	27.00	–	49.00	31.00	21.00	28.00
	**Number of VOCs with OAVs ≥ 1.0**	12	8	10	11	4	14	8	8	10

**Table 4:** Odor activity values (OAVs) of the volatile organic compounds detected in the different kiwi kefir-like beverages obtained from the third kefir grain passage (48–72 h).

No.	Compound	Bev1-72 h	Bev2-72 h	Bev3-72 h	Bev4-72 h	Bev5-72 h	Bev6-72 h	Bev7-72 h	Bev8-72 h	Bev9-72 h
	Organic acids									
1	Octanoic acid	–	–	–	–	–	–	0.38	–	–
	Alcohols									
2	1-Dodecanol	–	9.38	20.00	29.38	–	–	–	–	8.75
3	1-Hexadecanol	–	0.76	1.63	1.08	–	–	–	0.44	0.53
4	1-Hexanol	–	–	–	66.07	–	–	–	–	46.43
5	1-Octanol	–	–	–	–	–	–	–	–	3.42
6	1-Pentanol	63.00	40.58	25.58	16.67	39.25	62.67	41.25	21.75	47.75
7	1-Undecanol	–	0.37	0.39	0.70	–	1.09	0.30	0.79	0.39
8	2-Heptanol	–	–	–	–	–	–	–	–	1.99
9	2-Hexanol	–	–	–	–	–	–	–	–	–
10	2-Methyl-1-propanol	1.20	1.11	0.89	0.65	2.15	1.69	0.51	0.84	1.36
11	2-Nonanol	–	–	–	–	–	–	–	–	–
12	2-Phenylethanol	2.80	2.98	1.58	1.21	2.43	4.20	3.35	1.38	2.23
13	2-Undecanol	–	–	3.17	8.05	–	11.46	2.93	16.10	6.59
14	3-Methyl-1-pentanol	–	–	–	–	–	–	13.33	–	–
15	Furfuryl alcohol	0.10	0.03	0.04	0.06	–	0.05	0.06	0.22	–
	Aldehydes									
16	(E)-2-Hexenal	–	–	–	1.73	–	–	–	–	1.45
17	(E)-2-Nonenal	–	–	338.98	406.78	–	542.37	610.17	847.46	440.68
18	2-Methylbutanal	–	–	–	–	–	–	–	320.00	–
19	4-Hydroxy-3-methoxybenzaldehyde	10.38	4.34	5.85	7.36	–	4.91	6.60	–	7.55
20	5-Hydroxymethylfurfural	–	–	–	–	–	–	–	0.78	–
21	Benzaldehyde	0.81	0.32	0.41	0.49	–	0.56	0.71	–	0.57
22	Furfural	0.01	–	–	–	–	–	–	0.07	–
	Ketones									
23	2,6-Dimethyl-4-heptanone	7.73	7.91	9.36	12.36	11.64	12.09	6.73	2.55	12.82
24	2-Heptanone	–	–	–	–	–	–	–	–	–
25	4-Methyl-2-hexanone	–	–	–	–	–	–	–	–	–
	Esters									
26	2-Methylbutyl acetate	2.81	–	–	–	–	–	3.56	–	–
27	2-Phenylethyl acetate	0.96	0.52	0.68	–	0.56	0.76	0.76	–	–
28	Ethyl 3-phenylpropanoate	10.71	–	–	–	–	–	–	–	–
29	Ethyl butanoate	–	–	–	–	–	–	–	–	211.11
30	Ethyl decanoate	–	–	4.10	3.45	–	2.75	2.45	–	2.65
31	Ethyl dodecanoate	–	–	0.06	0.06	–	0.06	0.05	–	0.03
32	Ethyl hexadecanoate	–	–	0.19	0.15	–	–	–	–	–
33	Ethyl hexanoate	74.29	125.00	32.86	16.43	–	–	10.00	–	–
34	Ethyl octanoate	84.00	20.00	84.00	106.00	–	86.00	56.00	–	140.00
35	Hexyl acetate	–	–	0.19	–	–	–	–	–	–
36	Methyl benzoate	461.54	–	557.69	–	–	–	269.23	–	384.62
37	Pentyl acetate	–	1.16	11.40	–	7.44	10.00	–	–	–
	Furans									
38	2,5-Dimethyl-4-hydroxy-3(2H)-furanone	24.00	21.00	38.00	37.00	–	29.00	30.00	31.00	40.00
	**Number of VOCs with OAVs ≥ 1.0**	11	10	14	14	5	12	13	7	17

**Table 5:** Evolution of pH, microbial counts (lactic acid bacteria: LAB, acetic acid bacteria: AAB, and yeasts), organic acids (citric acid: CA, quinic acid: QA, lactic acid: LA, and acetic acid: AA), total sugars: TS, and alcohols (ethanol: EtOH, and glycerol: GOH) during the storage of the kiwi kefir-like beverages Bev7-48 h and Bev3-72 h at 4 °C over 7, 14, 21, and 28 days. The data are presented as means ± standard deviations from three independent experiments, each with two analytical replicates.

Bev7-48 h					
Variable	t = 0	t = 7 Days	t = 14 Days	t = 21 Days	t = 28 Days
pH	3.42 ± 0.01 ^A^	3.43 ± 0.01 ^A^	3.42 ± 0.02 ^A^	3.43 ± 0.02 ^A^	3.42 ± 0.01 ^A^
LAB (log CFU/mL)	6.36 ± 0.14 ^A^	6.41 ± 0.06 ^B,A^	6.39 ± 0.08 ^C,A,B^	6.29 ± 0.06 ^D,A,C^	6.32 ± 0.03 ^A,B,D^
AAB (log CFU/mL)	5.04 ± 0.01 ^A^	5.02 ± 0.01 ^B,A^	4.98 ± 0.03 ^C^	4.88 ± 0.05 ^D^	4.85 ± 0.06 ^E^
Yeasts (log CFU/mL)	6.30 ± 0.09 ^A^	6.50 ± 0.06 ^B^	6.68 ± 0.02 ^C^	6.71 ± 0.01 ^D,C^	6.71 ± 0.06 ^C,D^
CA (g/L)	9.73 ± 0.04 ^A^	8.94 ± 0.08 ^B^	8.51 ± 0.53 ^C,B^	7.49 ± 0.08 ^D^	6.46 ± 0.29 ^E^
QA (g/L)	7.18 ± 0.22 ^A^	7.00 ± 0.09 ^B^	6.44 ± 0.32 ^C^	6.30 ± 0.05 ^D,C^	5.86 ± 0.04 ^E^
LA (g/L)	0.64 ± 0.03 ^A^	0.70 ± 0.02 ^B^	0.73 ± 0.01 ^C^	0.75 ± 0.05 ^D,B,C^	0.77 ± 0.06 ^B,C,D^
AA (g/L)	0.40 ± 0.09 ^A^	0.52 ± 0.02 ^B^	0.56 ± 0.01 ^C^	0.64 ± 0.04 ^D^	0.71 ± 0.01 ^E^
EtOH (g/L)	1.26 ± 0.01 ^A^	1.39 ± 0.01 ^B^	1.49 ± 0.02 ^C^	1.68 ± 0.01 ^D^	1.71 ± 0.01 ^E^
GOH (g/L)	1.16 ± 0.14 ^A^	1.24 ± 0.06 ^B^	1.35 ± 0.08 ^C^	1.40 ± 0.06 ^D^	1.47 ± 0.03 ^E^
TS (g/L)	53.46 ± 0.01 ^A^	51.63 ± 0.01 ^B^	48.30 ± 0.03 ^C^	46.60 ± 0.05 ^D^	45.83 ± 0.06 ^E^
**Bev3-72 h**					
pH	3.15 ± 0.01 ^A^	3.15 ± 0.01 ^B,A^	3.14 ± 0.01 ^C,B^	3.13 ± 0.02 ^D,A,B,C^	3.13 ± 0.01 ^C,D^
LAB (log CFU/mL)	7.31 ± 0.04 ^A^	7.23 ± 0.10 ^B,A^	7.30 ± 0.08 ^C,A,B^	7.30 ± 0.02 ^A,B,C^	7.14 ± 0.07 ^B^
AAB (log CFU/mL)	4.39 ± 0.02 ^A^	4.08 ± 0.05 ^B^	3.95 ± 0.08 ^C^	3.91 ± 0.06 ^D^	3.91 ± 0.09 ^D^
Yeasts (log CFU/mL)	7.03 ± 0.03 ^A^	7.09 ± 0.03 ^B^	7.15 ± 0.05 ^C,B^	7.15 ± 0.02 ^D,C^	7.21 ± 0.02 ^E^
CA (g/L)	11.42 ± 0.36 ^A^	10.88 ± 0.06 ^B^	10.18 ± 0.25 ^C^	9.09 ± 0.11 ^D^	8.88 ± 0.08 ^E^
QA (g/L)	8.29 ± 0.21 ^A^	8.08 ± 0.11 ^B^	7.93 ± 0.11 ^B^	7.48 ± 0.12 ^C^	6.70 ± 0.48 ^D^
LA (g/L)	0.46 ± 0.05 ^A^	0.50 ± 0.02 ^B,A^	0.53 ± 0.01 ^C^	0.53 ± 0.02 ^D,B,C^	0.55 ± 0.02 ^C,D^
AA (g/L)	0.44 ± 0.03 ^A^	0.47 ± 0.01 ^B^	0.56 ± 0.02 ^C^	0.63 ± 0.03 ^D^	0.70 ± 0.06 ^E^
EtOH (g/L)	1.82 ± 0.00 ^A^	1.87 ± 0.00 ^B^	1.97 ± 0.01 ^C^	2.01 ± 0.02 ^D^	2.03 ± 0.01 ^E^
GOH (g/L)	1.43 ± 0.01 ^A^	1.51 ± 0.04 ^B^	1.56 ± 0.06 ^C^	1.61 ± 0.02 ^D^	1.63 ± 0.03 ^E^
TS (g/L)	56.40 ± 0.23 ^A^	54.47 ± 0.23 ^B^	52.60 ± 0.42 ^C^	50.80 ± 1.04 ^D^	46.03 ± 0.20 ^E^

Means within rows followed by the same letter are not significantly different after a significant ANOVA (*p* < 0.05).

## Data Availability

The data supporting the findings of this study are available within the article. Further inquiries can be directed to the corresponding author.

## Supplementary Materials

The following supporting information can be downloaded at https://www.mdpi.com/article/10.3390/foods14142502/s1, Table S1: Concentrations (mg/L) of volatile compounds identified in the unfermented kefir juice (UKJ) and in the beverages Bev1-24 h, Bev2-24 h, Bev3-24 h, Bev4-24 h, Bev5-24 h, Bev6-24 h, Bev7-24 h, Bev8-24 h, and Bev9-24 h. Values are expressed as means ± standard deviations, based on two independent experiments, each with two analytical replicates for the first eight beverages, and five independent experiments, each with two analytical replicates for the ninth beverage. The concentrations were determined using the HS-SPME 65 μm (PDMS/DVB, Fused Silica/SS)/GC-MS extraction method, with 3-octanol used as an internal standard. Table S2: Concentrations (mg/L) of volatile compounds identified in the unfermented kefir juice (UKJ) and in the beverages Bev1-48 h, Bev2-48 h, Bev3-48 h, Bev4-48 h, Bev5-48 h, Bev6-48 h, Bev7-48 h, Bev8-48 h, and Bev9-48 h. Values are expressed as means ± standard deviations, based on two independent experiments, each with two analytical replicates for the first eight beverages, and five independent experiments, each with two analytical replicates for the ninth beverage. The concentrations were determined using the HS-SPME 65 μm (PDMS/DVB, Fused Silica/SS)/GC-MS extraction method, with 3-octanol used as an internal standard. Table S3: Concentration (mg/L) of the volatile compounds identified in the unfermented kefir juice (UKJ) and in the beverages Bev1-72 h, Bev2-72 h, Bev3-72 h, Bev4-72 h, Bev5-72 h, Bev6-72 h, Bev7-72 h, Bev8-72 h, and Bev9-72 h. Values are expressed as means ± standard deviations, based on two independent experiments, each with two analytical replicates for the first eight beverages, and five independent experiments, each with two analytical replicates for the ninth beverage. The concentrations were determined using the HS-SPME 65 μm (PDMS/DVB, Fused Silica/SS)/GC-MS extraction method, with 3-octanol used as an internal standard. Table S4: The results of the experimental design and analysis of the significance of the proposed model for total concentration of alcohols at 24 h of fermentation. Table S5: The results of the experimental design and analysis of the significance of the proposed model for total concentration of alcohols at 48 h of fermentation. Table S6: The results of the experimental design and analysis of the significance of the proposed model for total concentration of alcohols at 72 h of fermentation. Table S7: The results of the experimental design and analysis of the significance of the proposed model for total concentration of aldehydes at 24 h of fermentation. Table S8: The results of the experimental design and analysis of the significance of the proposed model for total concentration of aldehydes at 48 h of fermentation. Table S9: The results of the experimental design and analysis of the significance of the proposed model for total concentration of aldehydes at 72 h of fermentation. Table S10: The results of the experimental design and analysis of the significance of the proposed model for total concentration of ketones at 24 h of fermentation. Table S11: The results of the experimental design and analysis of the significance of the proposed model for total concentration of ketones at 48 h of fermentation. Table S12: The results of the experimental design and analysis of the significance of the proposed model for total concentration of ketones at 72 h of fermentation. Table S13: The results of the experimental design and analysis of the significance of the proposed model for total concentration of esters at 24 h of fermentation. Table S14: The results of the experimental design and analysis of the significance of the proposed model for total concentration of esters at 48 h of fermentation. Table S15: The results of the experimental design and analysis of the significance of the proposed model for total concentration of esters at 72 h of fermentation. Table S16: The results of the experimental design and analysis of the significance of the proposed model for total concentration of furans at 24 h of fermentation. Table S17: The results of the experimental design and analysis of the significance of the proposed model for total concentration of furans at 48 h of fermentation. Table S18: The results of the experimental design and analysis of the significance of the proposed model for total concentration of furans at 72 h of fermentation. Table S19: The results of the experimental design and analysis of the significance of the proposed model for total concentration of “other compounds” at 24 h of fermentation. Table S20: The results of the experimental design and analysis of the significance of the proposed model for total concentration of “other compounds” at 48 h of fermentation. Table S21: The results of the experimental design and analysis of the significance of the proposed model for total concentration of “other compounds” at 72 h of fermentation. Table S22: The results of the experimental design and analysis of the significance of the proposed model for total concentration of volatile compounds ([TVOCs]) at 24 h of fermentation. Table S23: The results of the experimental design and analysis of the significance of the proposed model for total concentration of volatile compounds ([TVOCs]) at 48 h of fermentation. Table S24: The results of the experimental design and analysis of the significance of the proposed model for total concentration of volatile compounds ([TVOCs]) at 72 h of fermentation. Table S25: Total variance explained by the principal component analysis (PCA) of the 28 kiwifruit-based beverages, based on the initial concentrations of each VOC. Table S26: Total variance explained by principal component analysis (PCA) of the 28 kiwifruit-based beverages, based on the OAVs of VOCs with OAV ≥ 1.0. Table S27: Total variance explained by principal component analysis (PCA) of the 28 kiwifruit-based beverages, based on microbiological, chemical, and aromatic characteristics.
