# Supplementary material for: Part II—Volatile Profiles of Kiwi Kefir-like Beverages Influenced by the Amount of Inoculum, Shaking Rate, and Successive Kefir Grain Passages
Source: Foods. 2025 Jul 17;14(14):2502. doi: 10.3390/foods14142502 (PMC12294668; doi:10.3390/foods14142502)
Supplement: Supplementary file 1 [file foods-14-02502-s001.zip › foods-3693558-supplementary.pdf]

Table S1. Concentrations (mg/L) of volatile compounds identified in the unfermented kefir juice (UKJ) and in the beverages Bev1-24 h, Bev2-24 h, Bev3-24 h, Bev4-24 h, Bev5-24 h, Bev6-24 h, Bev7-24 h, Bev8-24 h, and Bev9-24 h. Values are expressed as means ± standard deviations, based on two independent experiments, each with two analytical replicates for the first eight beverages, and five independent experiments, each with two analytical replicates for the ninth beverage. The concentrations were determined using the HS-SPME 65 μm (PDMS/DVB, Fused Silica/SS) / GC-MS extraction method, with 3-octanol used as an internal standard.

No.	Compound	UKJ	Bev1-24 h	Bev2-24 h	Bev3-24 h	Bev4-24 h	Bev5-24 h	Bev6-24 h	Bev7-24 h	Bev8-24 h	Bev9-24 h
	Organic acids										
1	cis-9-Octadecenoic acid	N.d.	0.33 ± 0.07	N.d.	N.d.	N.d.	N.d.	N.d.	N.d.	N.d.	N.d.
2	Octanoic acid	N.d.	N.d.	N.d.	N.d.	N.d.	N.d.	N.d.	N.d.	N.d.	N.d.
	**Number of Organic Acids**	N.d.	1	N.d.	N.d.	N.d.	N.d.	N.d.	N.d.	N.d.	N.d.
	**Total concentration**	N.d.	0.33 ± 0.07	N.d.	N.d.	N.d.	N.d.	N.d.	N.d.	N.d.	N.d.
	Alcohols										
3	1-Dodecanol	N.d.	N.d.	N.d.	0.30 ± 0.00	0.57 ± 0.03	N.d.	N.d.	N.d.	N.d.	N.d.
4	1-Eicosanol	N.d.	N.d.	N.d.	N.d.	N.d.	N.d.	N.d.	N.d.	N.d.	N.d.
5	1-Heptadecanol	N.d.	N.d.	N.d.	N.d.	N.d.	N.d.	N.d.	N.d.	0.87 ± 0.02	N.d.
6	1-Hexadecanol	N.d.	0.42 ± 0.03	N.d.	N.d.	0.36 ± 0.08	N.d.	N.d.	0.13 ± 0.00	0.13 ± 0.00	0.78 ± 0.04
7	1-Hexanol	0.19 ± 0.00	N.d.	N.d.	N.d.	N.d.	0.16 ± 0.01	N.d.	0.13 ± 0.00	N.d.	N.d.
8	1-Octanol	N.d.	N.d.	N.d.	N.d.	N.d.	N.d.	N.d.	N.d.	N.d.	0.19 ± 0.04
9	1-Pentanol	N.d.	1.25 ± 0.05	0.62 ± 0.03	2.28 ± 0.00	0.13 ± 0.04	0.49 ± 0.02	2.14 ± 0.33	2.36 ± 0.39	0.18 ± 0.01	0.71 ± 0.04
10	1-Undecanol	N.d.	0.35 ± 0.00	0.05 ± 0.03	0.19 ± 0.05	0.22 ± 0.02	N.d.	0.50 ± 0.00	0.31 ± 0.04	0.62 ± 0.00	0.14 ± 0.01
11	2,6,8-Trimethyl-4-nonanol	N.d.	N.d.	N.d.	N.d.	N.d.	N.d.	N.d.	N.d.	N.d.	N.d.
12	2,7-Dimethyl-1-octanol	N.d.	N.d.	N.d.	N.d.	N.d.	N.d.	N.d.	N.d.	N.d.	0.10 ± 0.00
13	2-Butyl-1-octanol	N.d.	0.35 ± 0.00	0.47 ± 0.02	N.d.	0.38 ± 0.04	N.d.	1.18 ± 0.28	0.87 ± 0.02	0.20 ± 0.00	1.83 ± 0.26
14	2-Dodecanol	N.d.	0.17 ± 0.06	N.d.	0.30 ± 0.00	0.18 ± 0.06	N.d.	N.d.	0.21 ± 0.00	0.27 ± 0.01	0.17 ± 0.01
15	2-Heptanol	N.d.	N.d.	N.d.	N.d.	N.d.	N.d.	N.d.	N.d.	N.d.	N.d.
16	2-Hexanol	N.d.	0.19 ± 0.00	N.d.	0.40 ± 0.00	0.14 ± 0.02	N.d.	0.76 ± 0.09	0.13 ± 0.05	0.22 ± 0.08	0.29 ± 0.12
17	2-Methyl-1-propanol	N.d.	N.d.	N.d.	N.d.	N.d.	N.d.	1.14 ± 0.01	0.18 ± 0.00	N.d.	N.d.
18	2-Nonanol	N.d.	N.d.	N.d.	N.d.	0.13 ± 0.00	N.d.	N.d.	N.d.	N.d.	N.d.
19	2-Phenylethanol	0.16 ± 0.00	0.48 ± 0.06	0.84 ± 0.00	0.16 ± 0.00	0.02 ± 0.01	0.14 ± 0.05	0.41 ± 0.01	0.35 ± 0.01	0.12 ± 0.11	1.14 ± 0.72
20	2-Undecanol	N.d.	N.d.	N.d.	N.d.	0.05 ± 0.00	N.d.	0.27 ± 0.05	N.d.	0.24 ± 0.03	0.52 ± 0.02

21	3-Methyl-1-pentanol	N.d.	N.d.	N.d.	0.17 ± 0.04	N.d.	N.d.	N.d.	N.d.	N.d.	N.d.
22	7-Tetradecanol	N.d.	N.d.	N.d.	N.d.	N.d.	N.d.	N.d.	N.d.	N.d.	N.d.
23	E-10-Pentadecenol	N.d.	0.18 ± 0.00	N.d.	N.d.	N.d.	N.d.	0.40 ± 0.08	N.d.	N.d.	0.15 ± 0.01
24	E-2-Tridecen-1-ol	N.d.	N.d.	N.d.	N.d.	N.d.	N.d.	N.d.	N.d.	N.d.	N.d.
25	E-7-Tetradecenol	N.d.	N.d.	N.d.	N.d.	N.d.	N.d.	N.d.	N.d.	N.d.	N.d.
26	Furfuryl alcohol	0.99 ± 0.02	0.15 ± 0.02	0.11 ± 0.05	N.d.	0.15 ± 0.06	0.76 ± 0.33	N.d.	0.15 ± 0.04	0.66 ± 0.22	N.d.
	**Number of Alcohols**	3	9	5	7	11	4	8	10	10	11
	**Total concentration**	1.34 ± 0.02	3.55 ± 0.06	2.08 ± 0.05	3.79 ± 0.05	2.35 ± 0.08	1.54 ± 0.33	6.81 ± 0.33	4.82 ± 0.39	3.51 ± 0.22	6.02 ± 0.58
	Aldehydes										
27	(E)-2-Hexenal	N.d.	N.d.	N.d.	N.d.	N.d.	N.d.	N.d.	N.d.	N.d.	N.d.
28	(E)-2-Nonenal	N.d.	N.d.	N.d.	N.d.	0.06 ± 0.02	N.d.	0.50 ± 0.08	0.07 ± 0.00	N.d.	0.22 ± 0.04
29	(Z)-14-Methyl-8-hexadecenal	N.d.	N.d.	0.21 ± 0.02	N.d.	N.d.	N.d.	N.d.	N.d.	0.07 ± 0.02	N.d.
30	2,3-Dimethylpentanal	N.d.	N.d.	N.d.	N.d.	N.d.	N.d.	0.43 ± 0.04	N.d.	N.d.	N.d.
31	2-Ethyl-4-pentenal	N.d.	N.d.	N.d.	N.d.	N.d.	N.d.	N.d.	N.d.	N.d.	N.d.
32	2-Methylbutanal	N.d.	N.d.	N.d.	N.d.	N.d.	N.d.	N.d.	N.d.	0.04 ± 0.00	N.d.
33	4-Hydroxy-3-methoxybenzaldehyde	N.d.	0.25 ± 0.00	0.22 ± 0.01	0.45 ± 0.05	0.22 ± 0.06	N.d.	0.77 ± 0.01	0.28 ± 0.01	0.16 ± 0.02	0.35 ± 0.05
34	5-Hydroxymethylfurfural	N.d.	N.d.	N.d.	N.d.	N.d.	N.d.	N.d.	N.d.	0.46 ± 0.01	N.d.
35	Benzaldehyde	N.d.	0.25 ± 0.00	0.19 ± 0.01	0.50 ± 0.00	0.28 ± 0.00	N.d.	0.81 ± 0.04	0.40 ± 0.06	0.15 ± 0.02	0.62 ± 0.18
36	Furfural	N.d.	N.d.	N.d.	N.d.	N.d.	N.d.	N.d.	N.d.	0.16 ± 0.09	N.d.
	**Number of Aldehydes**	N.d.	2	3	2	3	N.d.	4	3	6	3
	**Total concentration**	N.d.	0.50 ± 0.00	0.62 ± 0.02	0.96 ± 0.05	0.56 ± 0.06	N.d.	2.51 ± 0.08	0.75 ± 0.06	1.04 ± 0.09	1.19 ± 0.18
	Ketones										
37	2,6-Dimethyl-4-heptanone	3.61 ± 0.66	1.06 ± 0.08	0.62 ± 0.01	1.22 ± 0.01	0.86 ± 0.07	1.02 ± 0.05	1.79 ± 0.11	0.94 ± 0.06	0.97 ± 0.08	1.89 ± 0.30
38	2-Heptanone	N.d.	N.d.	N.d.	0.25 ± 0.00	0.10 ± 0.07	N.d.	N.d.	N.d.	N.d.	N.d.
39	4-Methyl-2-hexanone	N.d.	N.d.	N.d.	N.d.	N.d.	N.d.	N.d.	N.d.	0.17 ± 0.00	N.d.
40	5-Methyl-2-heptanone	N.d.	N.d.	N.d.	N.d.	N.d.	N.d.	N.d.	N.d.	N.d.	N.d.
	**Number of Ketones**	1	1	1	2	2	1	1	1	2	1
	**Total concentration**	3.61 ± 0.66	1.06 ± 0.08	0.62 ± 0.01	1.46 ± 0.01	0.98 ± 0.07	1.02 ± 0.05	1.79 ± 0.11	0.94 ± 0.06	1.14 ± 0.08	1.89 ± 0,30
	Esters										

41	11,13-Dimethyltetradec-12-enyl acetate	N.d.	N.d.	N.d.	N.d.	N.d.	N.d.	N.d.	N.d.	N.d.	N.d.
42	1-Nonyl-2-(prop-2-enyl) oxalate	N.d.	N.d.	N.d.	N.d.	N.d.	N.d.	N.d.	0.18 ± 0.05	0.09 ± 0.01	N.d.
43	2-Methylbutyl acetate	N.d.	N.d.	N.d.	N.d.	N.d.	N.d.	N.d.	N.d.	N.d.	N.d.
44	2-Phenylethyl acetate	N.d.	0.09 ± 0.00	N.d.	N.d.	N.d.	N.d.	N.d.	0.08 ± 0.00	N.d.	N.d.
45	2-Tetradecyl 2-chloroacetate	N.d.	N.d.	0.03 ± 0.00	N.d.	N.d.	N.d.	N.d.	N.d.	N.d.	N.d.
46	3-Tridecyl methoxyacetate	N.d.	N.d.	N.d.	1.57 ± 0.02	0.11 ± 0.03	N.d.	N.d.	N.d.	0.64 ± 0.07	N.d.
47	Allyl pentadecyl oxalate	N.d.	N.d.	N.d.	N.d.	N.d.	N.d.	N.d.	N.d.	N.d.	N.d.
48	Benzyl oleate	N.d.	N.d.	N.d.	0.27 ± 0.04	N.d.	N.d.	0.38 ± 0.07	N.d.	N.d.	N.d.
49	Dodecan-2-yl prop-2-enoate	N.d.	0.09 ± 0.00	N.d.	N.d.	N.d.	N.d.	0.21 ± 0.10	0.09 ± 0.00	0.17 ± 0.00	0.25 ± 0.01
50	Dodecyl propyl oxalate	N.d.	N.d.	N.d.	N.d.	N.d.	N.d.	N.d.	N.d.	0.06 ± 0.01	N.d.
51	Ethyl 3-hydroxybutanoate	0.50 ± 0.30	0.05 ± 0.00	N.d.	0.06 ± 0.01	0.04 ± 0.01	0.29 ± 0.09	0.08 ± 0.00	0.02 ± 0.00	0.02 ± 0.01	0.07 ± 0.01
52	Ethyl 3-phenylpropanoate	N.d.	N.d.	N.d.	N.d.	N.d.	N.d.	N.d.	N.d.	N.d.	N.d.
53	Ethyl butanoate	0.71 ± 0.13	N.d.	N.d.	N.d.	N.d.	N.d.	N.d.	N.d.	N.d.	N.d.
54	Ethyl decanoate	N.d.	N.d.	N.d.	N.d.	0.02 ± 0.00	N.d.	N.d.	0.10 ± 0.01	N.d.	N.d.
55	Ethyl dodecanoate	N.d.	0.07 ± 0.02	N.d.	N.d.	N.d.	N.d.	N.d.	N.d.	N.d.	0.26 ± 0.07
56	Ethyl hexadecanoate	N.d.	N.d.	N.d.	N.d.	N.d.	N.d.	N.d.	N.d.	N.d.	N.d.
57	Ethyl hexanoate	0.60 ± 0.00	0.36 ± 0.00	N.d.	0.33 ± 0.02	0.13 ± 0.00	0.66 ± 0.05	N.d.	N.d.	N.d.	N.d.
58	Ethyl octanoate	N.d.	0.32 ± 0.00	0.19 ± 0.11	0.27 ± 0.09	0.88 ± 0.01	N.d.	0.60 ± 0.03	N.d.	N.d.	N.d.
59	Hexyl acetate	N.d.	N.d.	N.d.	N.d.	N.d.	N.d.	N.d.	N.d.	N.d.	N.d.
60	Methyl benzoate	N.d.	0.14 ± 0.07	N.d.	0.36 ± 0.00	0.16 ± 0.09	N.d.	N.d.	0.22 ± 0.01	N.d.	N.d.
61	Nonyl 2-acetyloxyacetate	N.d.	N.d.	N.d.	N.d.	N.d.	N.d.	N.d.	N.d.	N.d.	N.d.
62	Octadecyl chloroacetate	N.d.	0.59 ± 0.03	0.23 ± 0.01	0.18 ± 0.00	0.29 ± 0.11	N.d.	0.74 ± 0.00	N.d.	N.d.	0.49 ± 0.14
63	Pentyl 2,2-dimethylpropanoate	N.d.	N.d.	N.d.	N.d.	N.d.	N.d.	N.d.	N.d.	0.05 ± 0.01	0.10 ± 0.01
64	Pentyl acetate	N.d.	N.d.	N.d.	N.d.	N.d.	N.d.	N.d.	0.11 ± 0.01	N.d.	N.d.
	**Number of Esters**	3	8	3	7	7	2	5	7	6	5
	**Total concentration**	1.81 ± 0.30	1.72 ± 0.07	0.45 ± 0.11	3.04 ± 0.09	1.64 ± 0.11	0.95 ± 0.09	2.00 ± 0.10	0.80 ± 0.05	1.03 ± 0.07	1.17 ± 0.14
	Furans										

65	2,5-Dimethyl-4-hydroxy-3(2H)-furanone	N.d.	0.14 ± 0.00	0.20 ± 0.09	2.03 ± 0.01	0.27 ± 0.01	N.d.	0.51 ± 0.04	0.22 ± 0.01	0.10 ± 0.01	1.10 ± 0.04
66	4,4,5,5-Tetramethyl-dihydro-2(3H)-furanone	N.d.	N.d.	N.d.	N.d.	0.39 ± 0.03	N.d.	0.28 ± 0.02	N.d.	N.d.	0.71 ± 0.07
	**Number of Furans**	N.d.	1	1	1	2	N.d.	2	1	1	2
	**Total concentration**	N.d.	0.14 ± 0.00	0.20 ± 0.09	2.03 ± 0.01	0.66 ± 0.03	N.d.	0.79 ± 0.04	0.22 ± 0.01	0.10 ± 0.01	0.10 ± 0.07
67	***Other compounds***										
68	(Z)-3-Dodecene	0.13 ± 0.06	N.d.	N.d.	N.d.	N.d.	0.78 ± 0.02	N.d.	N.d.	N.d.	N.d.
68	Nonadecane	0.48 ± 0.00	N.d.	N.d.	N.d.	N.d.	0.48 ± 0.02	N.d.	N.d.	N.d.	N.d.
69	Tetradecane	0.33 ± 0.03	0.81 ± 0.01	N.d.	2.70 ± 0.14	0.82 ± 0.06	0.72 ± 0.17	1.90 ± 0.05	1.32 ± 0.01	0.38 ± 0.11	1.27 ± 0.61
70	7-Tetradecene	0.20 ± 0.00	N.d.	N.d.	N.d.	N.d.	N.d.	N.d.	N.d.	0.47 ± 0.07	N.d.
71	Hexadecane	N.d.	N.d.	N.d.	N.d.	0.97 ± 0.04	N.d.	N.d.	N.d.	N.d.	N.d.
72	2-Methyl-2-pentyl-oxirane	N.d.	N.d.	N.d.	N.d.	N.d.	N.d.	N.d.	N.d.	N.d.	0.10 ± 0.01
73	1-Decylsulfonyldecane	N.d.	N.d.	N.d.	N.d.	N.d.	N.d.	N.d.	N.d.	N.d.	N.d.
74	Benzoyl bromide	N.d.	1.75 ± 0.00	1.12 ± 0.22	1.31 ± 0.00	0.94 ± 0.00	3.11 ± 1.05	1.92 ± 0.03	1.35 ± 0.00	0.53 ± 0.04	1.38 ± 0.46
75	Methanesulfonyl azide	N.d.	N.d.	N.d.	N.d.	N.d.	N.d.	N.d.	N.d.	N.d.	N.d.
76	Methoxyphenyl oxime	N.d.	0.09 ± 0.01	N.d.	N.d.	0.26 ± 0.01	N.d.	N.d.	N.d.	N.d.	0.46 ± 0.04
77	O-Decyl-hydroxylamine	0.28 ± 0.00	N.d.	0.27 ± 0.01	N.d.	N.d.	0.29 ± 0.01	N.d.	N.d.	N.d.	N.d.
78	n-Decyl sulfone	N.d.	N.d.	N.d.	N.d.	N.d.	N.d.	N.d.	N.d.	N.d.	N.d.
79	1,2,3-Trimethylbenzene	N.d.	1.10 ± 0.04	0.76 ± 0.11	1.64 ± 0.02	0.80 ± 0.09	N.d.	2.70 ± 0.00	1.43 ± 0.00	1.12 ± 0.08	1.66 ± 0.15
80	1,2,4-Trimethylbenzene	0.70 ± 0.59	N.d.	N.d.	N.d.	N.d.	0.55 ± 0.08	N.d.	N.d.	N.d.	N.d.
81	2,4-Dimethyl-2-pentene	N.d.	N.d.	N.d.	N.d.	0.14 ± 0.09	N.d.	N.d.	N.d.	N.d.	N.d.
82	3,5-Dimethyl-1-hexene	0.24 ± 0.00	N.d.	N.d.	N.d.	N.d.	0.21 ± 0.01	N.d.	N.d.	N.d.	N.d.
	**Number of Other Compounds**	7	4	3	3	6	7	3	3	4	5
	**Total concentration**	2.36 ± 0.59	3.74 ± 0.04	2.15 ± 0.22	5.65 ± 0.14	3.93 ± 0.09	6.15 ± 1.05	6.52 ± 0.05	4.10 ± 0.01	2.49 ± 0.11	4.86 ± 0.61
	**Number of Volatile Compounds**	14	26	16	22	31	14	23	25	29	27
	**Total concentration of Volatile Compounds**	9.14 ± 0.43	11.06 ± 0.31	6.12 ± 0.20	16.94 ± 0.53	10.07 ± 0.24	9.66 ± 0.39	20.42 ± 0.55	11.59 ± 0.38	9.32 ± 0.23	16.95 ± 0.43

* N.d.: not detected.

Table S2. Concentrations (mg/L) of volatile compounds identified in the unfermented kefir juice (UKJ) and in the beverages Bev1-48 h, Bev2-48 h, Bev3-48 h, Bev4-48 h, Bev5-48 h, Bev6-48 h, Bev7-48 h, Bev8-48 h, and Bev9-48 h. Values are expressed as means ± standard deviations, based on two independent experiments, each with two analytical replicates for the first eight beverages, and five independent experiments, each with two analytical replicates for the ninth beverage. The concentrations were determined using the HS-SPME 65 µm (PDMS/DVB, Fused Silica/SS) / GC-MS extraction method, with 3-octanol used as an internal standard.

No.	Compound	Bev1-48 h	Bev2-48 h	Bev3-48 h	Bev4-48 h	Bev5-48 h	Bev6-48 h	Bev7-48 h	Bev8-48 h	Bev9-48 h
	Organic acids									
1	cis-9-Octadecenoic acid	N.d.	N.d.	N.d.	N.d.	N.d.	N.d.	N.d.	N.d.	N.d.
2	Octanoic acid	N.d.	N.d.	N.d.	N.d.	N.d.	N.d.	N.d.	N.d.	N.d.
	**Number of Organic Acids**	N.d.	N.d.	N.d.	N.d.	N.d.	N.d.	N.d.	N.d.	N.d.
	**Total concentration**	N.d.	N.d.	N.d.	N.d.	N.d.	N.d.	N.d.	N.d.	N.d.
	Alcohols									
3	1-Dodecanol	0.10 ± 0.00	N.d.	0.62 ± 0.03	0.31 ± 0.02	N.d.	N.d.	N.d.	N.d.	0.10 ± 0.01
4	1-Eicosanol	0.09 ± 0.01	N.d.	N.d.	N.d.	N.d.	N.d.	N.d.	N.d.	N.d.
5	1-Heptadecanol	N.d.	N.d.	N.d.	N.d.	N.d.	N.d.	N.d.	N.d.	N.d.
6	1-Hexadecanol	0.43 ± 0.16	N.d.	0.34 ± 0.05	0.26 ± 0.03	N.d.	N.d.	N.d.	0.31 ± 0.03	0.51 ± 0.03
7	1-Hexanol	N.d.	0.03 ± 0.00	N.d.	0.15 ± 0.01	N.d.	N.d.	N.d.	N.d.	N.d.
8	1-Octanol	N.d.	N.d.	N.d.	N.d.	N.d.	N.d.	N.d.	N.d.	N.d.
9	1-Pentanol	3.15 ± 0.01	1.43 ± 0.01	1.99 ± 0.01	0.35 ± 0.01	1.44 ± 0.06	3.81 ± 0.05	3.09 ± 0.06	1.63 ± 0.13	4.56 ± 0.27
10	1-Undecanol	0.19 ± 0.02	0.10 ± 0.01	0.13 ± 0.01	N.d.	N.d.	1.22 ± 0.01	0.09 ± 0.01	0.24 ± 0.02	0.28 ± 0.02
11	2,6,8-Trimethyl-4-nonanol	N.d.	N.d.	N.d.	N.d.	N.d.	N.d.	N.d.	N.d.	N.d.
12	2,7-Dimethyl-1-octanol	N.d.	N.d.	N.d.	N.d.	N.d.	N.d.	N.d.	N.d.	N.d.
13	2-Butyl-1-octanol	0.25 ± 0.00	0.38 ± 0.03	1.65 ± 0.06	0.15 ± 0.01	N.d.	1.71 ± 0.07	0.44 ± 0.03	0.56 ± 0.10	1.00 ± 0.06
14	2-Dodecanol	0.11 ± 0.03	N.d.	0.23 ± 0.02	0.21 ± 0.01	N.d.	0.43 ± 0.04	0.12 ± 0.01	0.13 ± 0.04	0.20 ± 0.03
15	2-Heptanol	N.d.	N.d.	N.d.	N.d.	N.d.	N.d.	N.d.	N.d.	0.17 ± 0.01
16	2-Hexanol	0.06 ± 0.00	0.06 ± 0.01	0.33 ± 0.03	0.17 ± 0.01	N.d.	0.24 ± 0.01	0.23 ± 0.02	N.d.	0.21 ± 0.01
17	2-Methyl-1-propanol	0.32 ± 0.15	0.20 ± 0.01	0.29 ± 0.01	N.d.	N.d.	1.05 ± 0.03	0.13 ± 0.01	0.31 ± 0.03	0.27 ± 0.08
18	2-Nonanol	N.d.	N.d.	N.d.	N.d.	N.d.	N.d.	N.d.	N.d.	N.d.
19	2-Phenylethanol	0.76 ± 0.00	0.61 ± 0.03	0.43 ± 0.02	0.06 ± 0.00	0.78 ± 0.03	1.26 ± 0.05	0.52 ± 0.00	0.60 ± 0.02	1.08 ± 0.35
20	2-Undecanol	0.08 ± 0.00	N.d.	N.d.	0.27 ± 0.03	N.d.	0.28 ± 0.02	0.07 ± 0.01	0.10 ± 0.00	0.19 ± 0.03

21	3-Methyl-1-pentanol	N.d.	N.d.	N.d.	N.d.	N.d.	N.d.	N.d.	N.d.	N.d.
22	7-Tetradecanol	N.d.	N.d.	0.21 ± 0.01	N.d.	N.d.	N.d.	N.d.	N.d.	N.d.
23	E-10-Pentadecenol	0.15 ± 0.01	N.d.	N.d.	N.d.	N.d.	0.36 ± 0.03	N.d.	N.d.	0.26 ± 0.02
24	E-2-Tridecen-1-ol	N.d.	N.d.	N.d.	N.d.	0.20 ± 0.01	N.d.	N.d.	N.d.	N.d.
25	E-7-Tetradecenol	N.d.	0.06 ± 0.00	N.d.	N.d.	N.d.	N.d.	N.d.	N.d.	N.d.
26	Furfuryl alcohol	0.19 ± 0.01	N.d.	N.d.	0.15 ± 0.01	N.d.	0.20 ± 0.01	0.12 ± 0.01	N.d.	N.d.
	**Number of Alcohols**	13	8	10	10	3	10	9	8	12
	**Total concentration**	5.87 ± 0.16	2.86 ± 0.03	6.22 ± 0.06	2.08 ± 0.03	2.42 ± 0.06	10.57 ± 0.07	4.81 ± 0.06	3.89 ± 0.13	9.29 ± 0.27
	Aldehydes									
27	(E)-2-Hexenal	N.d.	N.d.	N.d.	N.d.	N.d.	N.d.	N.d.	N.d.	0.11 ± 0.01
28	(E)-2-Nonenal	N.d.	N.d.	0.37 ± 0.02	0.08 ± 0.01	N.d.	0.43 ± 0.05	N.d.	0.10 ± 0.01	0.11 ± 0.01
29	(Z)-14-Methyl-8-hexadecenal	0.41 ± 0.00	0.17 ± 0.00	N.d.	N.d.	N.d.	N.d.	N.d.	N.d.	N.d.
30	2,3-Dimethylpentanal	N.d.	0.17 ± 0.03	0.60 ± 0.06	N.d.	N.d.	N.d.	0.76 ± 0.11	0.21 ± 0.03	0.29 ± 0.03
31	2-Ethyl-4-pentenal	N.d.	N.d.	N.d.	N.d.	N.d.	N.d.	N.d.	N.d.	N.d.
32	2-Methylbutanal	N.d.	N.d.	N.d.	N.d.	0.06 ± 0.01	N.d.	N.d.	N.d.	N.d.
33	4-Hydroxy-3-methoxybenzaldehyde	0.13 ± 0.03	0.20 ± 0.04	0.61 ± 0.01	0.29 ± 0.02	N.d.	0.33 ± 0.01	0.21 ± 0.02	0.25 ± 0.02	0.28 ± 0.05
34	5-Hydroxymethylfurfural	N.d.	N.d.	N.d.	N.d.	N.d.	N.d.	N.d.	N.d.	N.d.
35	Benzaldehyde	0.13 ± 0.01	0.16 ± 0.01	0.58 ± 0.09	0.29 ± 0.02	N.d.	0.53 ± 0.04	0.22 ± 0.01	0.23 ± 0.02	0.31 ± 0.03
36	Furfural	N.d.	N.d.	N.d.	N.d.	N.d.	N.d.	N.d.	N.d.	N.d.
	**Number of Aldehydes**	3	4	4	3	N.d.	3	3	4	5
	**Total concentration**	0.67 ± 0.03	0.71 ± 0.04	2.16 ± 0.09	0.67 ± 0.06	N.d.	1.29 ± 0.05	1.19 ± 0.11	0.80 ± 0.03	1.10 ± 0.05
	Ketones									
37	2,6-Dimethyl-4-heptanone	0.64 ± 0.05	0.46 ± 0.02	0.41 ± 0.03	1.05 ± 0.13	1.32 ± 0.11	1.18 ± 0.08	0.47 ± 0.02	0.67 ± 0.02	0.83 ± 0.04
38	2-Heptanone	N.d.	N.d.	N.d.	0.21 ± 0.04	N.d.	N.d.	N.d.	N.d.	N.d.
39	4-Methyl-2-hexanone	N.d.	N.d.	N.d.	N.d.	N.d.	N.d.	N.d.	N.d.	N.d.
40	5-Methyl-2-heptanone	N.d.	N.d.	N.d.	N.d.	N.d.	N.d.	N.d.	N.d.	N.d.
	**Number of Ketones**	1	1	1	2	1	1	1	1	1
	**Total concentration**	0.64 ± 0.05	0.46 ± 0.02	0.41 ± 0.03	1.26 ± 0.13	1.38 ± 0.11	1.18 ± 0.08	0.47 ± 0.02	0.67 ± 0.02	0.83 ± 0.04
	Esters									

41	11,13-Dimethyltetradec-12-enyl acetate	N.d.	N.d.	N.d.	N.d.	N.d.	N.d.	N.d.	N.d.	N.d.
42	1-Nonyl-2-(prop-2-enyl) oxalate	N.d.	N.d.	N.d.	N.d.	N.d.	N.d.	N.d.	0.08 ± 0.01	0.57 ± 0.03
43	2-Methylbutyl acetate	0.22 ± 0.00	N.d.	0.15 ± 0.01	N.d.	N.d.	N.d.	N.d.	N.d.	N.d.
44	2-Phenylethyl acetate	0.03 ± 0.01	N.d.	N.d.	N.d.	0.17 ± 0.03	0.30 ± 0.00	0.05 ± 0.00	N.d.	N.d.
45	2-Tetradecyl 2-chloroacetate	N.d.	0.09 ± 0.00	N.d.	0.15 ± 0.01	N.d.	N.d.	N.d.	N.d.	N.d.
46	3-Tridecyl methoxyacetate	N.d.	N.d.	0.32 ± 0.02	0.15 ± 0.01	N.d.	N.d.	N.d.	0.90 ± 0.01	N.d.
47	Allyl pentadecyl oxalate	N.d.	0.46 ± 0.06	N.d.	N.d.	0.20 ± 0.01	N.d.	N.d.	N.d.	N.d.
48	Benzyl oleate	0.08 ± 0.01	N.d.	0.28 ± 0.03	N.d.	N.d.	N.d.	N.d.	N.d.	0.18 ± 0.01
49	Dodecan-2-yl prop-2-enoate	N.d.	N.d.	0.20 ± 0.01	N.d.	N.d.	N.d.	N.d.	0.08 ± 0.00	N.d.
50	Dodecyl propyl oxalate	N.d.	N.d.	N.d.	N.d.	N.d.	N.d.	0.10 ± 0.01	N.d.	N.d.
51	Ethyl 3-hydroxybutanoate	0.01 ± 0.00	0.02 ± 0.00	0.09 ± 0.02	0.05 ± 0.01	0.06 ± 0.01	0.13 ± 0.02	0.03 ± 0.00	0.03 ± 0.00	0.08 ± 0.03
52	Ethyl 3-phenylpropanoate	N.d.	N.d.	N.d.	N.d.	N.d.	N.d.	N.d.	N.d.	N.d.
53	Ethyl butanoate	N.d.	N.d.	N.d.	N.d.	N.d.	N.d.	N.d.	N.d.	N.d.
54	Ethyl decanoate	N.d.	N.d.	0.27 ± 0.02	N.d.	N.d.	1.22 ± 0.04	0.23 ± 0.01	N.d.	0.18 ± 0.01
55	Ethyl dodecanoate	N.d.	N.d.	0.10 ± 0.02	N.d.	N.d.	0.57 ± 0.01	0.14 ± 0.02	N.d.	0.20 ± 0.06
56	Ethyl hexadecanoate	N.d.	N.d.	N.d.	N.d.	N.d.	N.d.	N.d.	N.d.	N.d.
57	Ethyl hexanoate	1.03 ± 0.03	N.d.	0.41 ± 0.06	0.13 ± 0.01	N.d.	0.59 ± 0.01	0.12 ± 0.04	N.d.	N.d.
58	Ethyl octanoate	0.12 ± 0.00	0.11 ± 0.01	0.28 ± 0.04	0.49 ± 0.07	N.d.	0.43 ± 0.01	0.46 ± 0.06	N.d.	0.26 ± 0.03
59	Hexyl acetate	N.d.	N.d.	N.d.	N.d.	N.d.	N.d.	N.d.	N.d.	N.d.
60	Methyl benzoate	0.19 ± 0.03	0.07 ± 0.01	0.15 ± 0.04	N.d.	N.d.	N.d.	N.d.	0.11 ± 0.00	N.d.
61	Nonyl 2-acetyloxyacetate	N.d.	N.d.	N.d.	N.d.	N.d.	N.d.	N.d.	N.d.	N.d.
62	Octadecyl chloroacetate	0.27 ± 0.02	0.05 ± 0.01	0.25 ± 0.03	0.44 ± 0.06	N.d.	1.03 ± 0.00	0.22 ± 0.01	N.d.	0.24 ± 0.01
63	Pentyl 2,2-dimethylpropanoate	N.d.	N.d.	0.11 ± 0.01	0.06 ± 0.00	N.d.	N.d.	N.d.	0.05 ± 0.00	N.d.
64	Pentyl acetate	0.06 ± 0.00	N.d.	N.d.	N.d.	N.d.	0.36 ± 0.04	N.d.	N.d.	N.d.
	**Number of Esters**	9	6	12	7	3	8	8	6	7
	**Total concentration**	2.01 ± 0.03	0.81 ± 0.06	2.60 ± 0.06	1.47 ± 0.07	0.43 ± 0.03	4.62 ± 0.04	1.35 ± 0.06	1.26 ± 0.01	1.73 ± 0.06
	Furans									

65	2,5-Dimethyl-4-hydroxy-3(2H)-furanone	0.10 ± 0.01	0.16 ± 0.01	0.58 ± 0.03	0.27 ± 0.01	N.d.	0.49 ± 0.02	0.31 ± 0.04	0.21 ± 0.01	0.28 ± 0.01
66	4,4,5,5-Tetramethyl-dihydro-2(3H)-furanone	N.d.	N.d.	N.d.	N.d.	N.d.	0.22 ± 0.01	N.d.	N.d.	0.23 ± 0.05
	**Number of Furans**	1	1	1	2	N.d.	2	1	1	2
	**Total concentration**	0.10 ± 0.01	0.16 ± 0.01	0.58 ± 0.03	0.27 ± 0.01	N.d.	0.71 ± 0.02	0.31 ± 0.04	0.21 ± 0.01	0.51 ± 0.05
	Other compounds									
67	(Z)-3-Dodecene	N.d.	N.d.	N.d.	N.d.	0.68 ± 0.01	N.d.	N.d.	N.d.	N.d.
68	Nonadecane	N.d.	N.d.	N.d.	N.d.	0.85 ± 0.08	N.d.	N.d.	N.d.	N.d.
69	Tetradecane	1.47 ± 0.04	N.d.	0.49 ± 0.02	0.88 ± 0.07	0.66 ± 0.02	0.31 ± 0.02	0.73 ± 0.05	0.43 ± 0.02	1.06 ± 0.40
70	7-Tetradecene	N.d.	N.d.	N.d.	N.d.	N.d.	N.d.	N.d.	N.d.	N.d.
71	Hexadecane	N.d.	N.d.	N.d.	1.32 ± 0.03	N.d.	N.d.	N.d.	N.d.	0.92 ± 0.05
72	2-Methyl-2-pentyl-oxirane	N.d.	N.d.	N.d.	0.06 ± 0.00	N.d.	N.d.	N.d.	0.04 ± 0.00	N.d.
73	1-Decylsulfonyldecane	N.d.	N.d.	0.14 ± 0.01	N.d.	N.d.	N.d.	N.d.	N.d.	N.d.
74	Benzoyl bromide	0.34 ± 0.02	0.74 ± 0.10	0.36 ± 0.05	1.30 ± 0.06	3.12 ± 0.03	1.05 ± 0.04	0.70 ± 0.02	0.66 ± 0.03	0.91 ± 0.14
75	Methanesulfonyl azide	N.d.	N.d.	N.d.	N.d.	0.23 ± 0.00	N.d.	N.d.	N.d.	N.d.
76	Methoxyphenyl oxime	N.d.	N.d.	N.d.	N.d.	N.d.	N.d.	0.18 ± 0.00	N.d.	0.32 ± 0.02
77	O-Decyl-hydroxylamine	N.d.	N.d.	N.d.	N.d.	N.d.	N.d.	N.d.	N.d.	N.d.
78	n-Decyl sulfone	N.d.	N.d.	N.d.	N.d.	N.d.	N.d.	N.d.	N.d.	N.d.
79	1,2,3-Trimethylbenzene	0.45 ± 0.08	0.50 ± 0.04	0.44 ± 0.05	1.37 ± 0.06	N.d.	1.05 ± 0.02	0.65 ± 0.02	0.85 ± 0.06	1.04 ± 0.15
80	1,2,4-Trimethylbenzene	N.d.	N.d.	N.d.	N.d.	0.40 ± 0.01	N.d.	N.d.	N.d.	N.d.
81	2,4-Dimethyl-2-pentene	N.d.	N.d.	N.d.	N.d.	N.d.	N.d.	N.d.	N.d.	N.d.
82	3,5-Dimethyl-1-hexene	N.d.	N.d.	N.d.	N.d.	N.d.	0.59 ± 0.02	N.d.	N.d.	N.d.
	**Number of Other Compounds**	3	2	4	5	6	4	4	4	5
	**Total concentration**	2.26 ± 0.08	1.24 ± 0.10	1.42 ± 0.05	4.92 ± 0.07	5.94 ± 0.08	3.00 ± 0.04	2.26 ± 0.05	1.99 ± 0.06	4.25 ± 0.40
	**Number of Volatile Compounds**	30	22	32	28	14	28	26	24	32

	**Total concentration of Volatile Compounds**	11.56 ± 0.41	6.23 ± 0.21	13.41 ± 0.32	10.67 ± 0.29	10.17 ± 0.43	21.37 ± 0.55	10.39 ± 0.38	8.78 ± 0.26	17.23 ± 0.56

* N.d.: not detected.

Table S3. Concentrations (mg/L) of volatile compounds identified in the unfermented kefir juice (UKJ) and in the beverages Bev1-72 h, Bev2-72 h, Bev3-72 h, Bev4-72 h, Bev5-72 h, Bev6-72 h, Bev7-72 h, Bev8-72 h, and Bev9-72 h. Values are expressed as means ± standard deviations, based on two independent experiments, each with two analytical replicates for the first eight beverages, and five independent experiments, each with two analytical replicates for the ninth beverage. The concentrations were determined using the HS-SPME 65 μm (PDMS/DVB, Fused Silica/SS) / GC-MS extraction method, with 3-octanol used as an internal standard.

No.	Compound	Bev1-72 h	Bev2-72 h	Bev3-72 h	Bev4-72 h	Bev5-72 h	Bev6-72 h	Bev7-72 h	Bev8-72 h	Bev9-72 h
	Organic acids									
1	cis-9-Octadecenoic acid	N.d.	N.d.	N.d.	N.d.	N.d.	N.d.	N.d.	N.d.	N.d.
2	Octanoic acid	N.d.	N.d.	N.d.	N.d.	N.d.	N.d.	0.19 ± 0.01	N.d.	N.d.
	**Number of Organic Acids**	N.d.	N.d.	N.d.	N.d.	N.d.	N.d.	1	N.d.	N.d.
	**Total concentration**	N.d.	N.d.	N.d.	N.d.	N.d.	N.d.	0.19 ± 0.01	N.d.	N.d.
	Alcohols									
3	1-Dodecanol	N.d.	0.15 ± 0.01	0.32 ± 0.03	0.47 ± 0.01	N.d.	N.d.	N.d.	N.d.	0.14 ± 0.01
4	1-Eicosanol	N.d.	0.23 ± 0.01	N.d.	N.d.	N.d.	N.d.	N.d.	N.d.	N.d.
5	1-Heptadecanol	N.d.	N.d.	N.d.	N.d.	N.d.	1.30 ± 0.03	N.d.	N.d.	N.d.
6	1-Hexadecanol	N.d.	0.57 ± 0.02	1.22 ± 0.04	0.81 ± 0.02	N.d.	N.d.	N.d.	0.33 ± 0.01	0.40 ± 0.03
7	1-Hexanol	N.d.	N.d.	N.d.	0.37 ± 0.01	N.d.	N.d.	N.d.	N.d.	0.26 ± 0.13
8	1-Octanol	N.d.	N.d.	N.d.	N.d.	N.d.	N.d.	N.d.	N.d.	0.43 ± 0.03
9	1-Pentanol	7.56 ± 0.11	4.87 ± 0.13	3.07 ± 0.05	2.00 ± 0.01	4.71 ± 0.06	7.52 ± 0.14	4.95 ± 0.05	2.61 ± 0.01	5.73 ± 0.49
10	1-Undecanol	N.d.	0.26 ± 0.01	0.27 ± 0.01	0.49 ± 0.01	N.d.	0.76 ± 0.03	0.21 ± 0.01	0.55 ± 0.01	0.27 ± 0.02
11	2,6,8-Trimethyl-4-nonanol	N.d.	N.d.	N.d.	N.d.	N.d.	N.d.	0.12 ± 0.01	N.d.	N.d.
12	2,7-Dimethyl-1-octanol	N.d.	N.d.	N.d.	N.d.	N.d.	N.d.	N.d.	N.d.	0.42 ± 0.03
13	2-Butyl-1-octanol	0.41 ± 0.03	1.22 ± 0.01	0.49 ± 0.01	0.25 ± 0.01	N.d.	2.33 ± 0.06	0.73 ± 0.02	0.92 ± 0.05	1.64 ± 0.16
14	2-Dodecanol	N.d.	0.10 ± 0.01	0.16 ± 0.01	0.12 ± 0.01	N.d.	0.38 ± 0.01	N.d.	0.42 ± 0.01	0.24 ± 0.01
15	2-Heptanol	N.d.	N.d.	N.d.	N.d.	N.d.	N.d.	N.d.	N.d.	0.13 ± 0.01
16	2-Hexanol	N.d.	0.15 ± 0.01	0.23 ± 0.01	0.24 ± 0.01	N.d.	0.07 ± 0.00	0.11 ± 0.01	N.d.	0.22 ± 0.03
17	2-Methyl-1-propanol	0.66 ± 0.03	0.61 ± 0.02	0.49 ± 0.01	0.36 ± 0.00	1.18 ± 0.02	0.93 ± 0.01	0.28 ± 0.01	0.46 ± 0.01	0.75 ± 0.10
18	2-Nonanol	N.d.	N.d.	N.d.	N.d.	N.d.	N.d.	N.d.	N.d.	N.d.
19	2-Phenylethanol	1.58 ± 0.03	1.68 ± 0.02	0.89 ± 0.02	0.68 ± 0.03	1.37 ± 0.02	2.37 ± 0.01	1.89 ± 0.05	0.78 ± 0.09	1.26 ± 0.07
20	2-Undecanol	N.d.	N.d.	0.13 ± 0.01	0.33 ± 0.01	N.d.	0.47 ± 0.02	0.12 ± 0.01	0.66 ± 0.00	0.27 ± 0.02

21	3-Methyl-1-pentanol	N.d.	N.d.	N.d.	N.d.	N.d.	N.d.	0.10 ± 0.01	N.d.	N.d.
22	7-Tetradecanol	N.d.	0.22 ± 0.02	0.15 ± 0.01	N.d.	N.d.	N.d.	N.d.	N.d.	N.d.
23	E-10-Pentadecenol	N.d.	N.d.	N.d.	N.d.	N.d.	0.33 ± 0.01	N.d.	N.d.	0.23 ± 0.02
24	E-2-Tridecen-1-ol	N.d.	N.d.	N.d.	N.d.	0.48 ± 0.08	N.d.	N.d.	N.d.	N.d.
25	E-7-Tetradecenol	N.d.	N.d.	N.d.	N.d.	N.d.	N.d.	N.d.	N.d.	N.d.
26	Furfuryl alcohol	0.44 ± 0.01	0.14 ± 0.01	0.18 ± 0.02	0.25 ± 0.01	N.d.	0.21 ± 0.01	0.28 ± 0.01	1.01 ± 0.04	N.d.
	**Number of Alcohols**	5	12	12	12	4	11	10	9	15
	**Total concentration**	10.66 ± 0.11	10.21 ± 0.13	7.60 ± 0.05	6.38 ± 0.03	7.74 ± 0.08	16.66 ± 0.14	8.80 ± 0.05	7.75 ± 0.09	12.38 ± 0.49
	Aldehydes									
27	(E)-2-Hexenal	N.d.	N.d.	N.d.	0.19 ± 0.01	N.d.	N.d.	N.d.	N.d.	0.16 ± 0.01
28	(E)-2-Nonenal	N.d.	N.d.	0.10 ± 0.01	0.12 ± 0.01	N.d.	0.16 ± 0.04	0.18 ± 0.02	0.25 ± 0.01	0.13 ± 0.03
29	(Z)-14-Methyl-8-hexadecenal	N.d.	N.d.	N.d.	N.d.	N.d.	N.d.	N.d.	N.d.	N.d.
30	2,3-Dimethylpentanal	N.d.	0.25 ± 0.02	N.d.	N.d.	N.d.	N.d.	N.d.	0.51 ± 0.04	0.68 ± 0.12
31	2-Ethyl-4-pentenal	N.d.	N.d.	N.d.	N.d.	N.d.	N.d.	0.20 ± 0.01	N.d.	N.d.
32	2-Methylbutanal	N.d.	N.d.	N.d.	N.d.	N.d.	N.d.	N.d.	0.32 ± 0.01	N.d.
33	4-Hydroxy-3-methoxybenzaldehyde	0.55 ± 0.03	0.23 ± 0.01	0.31 ± 0.02	0.39 ± 0.01	N.d.	0.26 ± 0.01	0.35 ± 0.04	N.d.	0.40 ± 0.04
34	5-Hydroxymethylfurfural	N.d.	N.d.	N.d.	N.d.	N.d.	N.d.	N.d.	0.78 ± 0.05	N.d.
35	Benzaldehyde	0.61 ± 0.03	0.24 ± 0.01	0.31 ± 0.01	0.37 ± 0.00	N.d.	0.42 ± 0.01	0.53 ± 0.03	N.d.	0.43 ± 0.07
36	Furfural	0.17 ± 0.01	N.d.	N.d.	N.d.	N.d.	N.d.	N.d.	0.92 ± 0.07	N.d.
	**Number of Aldehydes**	3	3	3	4	N.d.	3	4	5	5
	**Total concentration**	1.33 ± 0.03	0.71 ± 0.02	0.71 ± 0.02	1.06 ± 0.01	N.d.	0.84± 0.04	1.26 ± 0.04	2.78 ± 0.07	1.81 ± 0.12
	Ketones									
37	2,6-Dimethyl-4-heptanone	0.85 ± 0.01	0.87 ± 0.04	1.03 ± 0.02	1.36 ± 0.03	1.28 ± 0.05	1.33 ± 0.01	0.74 ± 0.01	0.28 ± 0.01	1.41 ± 0.20
38	2-Heptanone	N.d.	N.d.	N.d.	N.d.	N.d.	N.d.	N.d.	N.d.	N.d.
39	4-Methyl-2-hexanone	N.d.	N.d.	N.d.	N.d.	N.d.	N.d.	N.d.	N.d.	N.d.
40	5-Methyl-2-heptanone	N.d.	N.d.	N.d.	0.53 ± 0.01	N.d.	N.d.	N.d.	N.d.	N.d.
	**Number of Ketones**	1	1	1	2	1	1	1	1	1
	**Total concentration**	0.85 ± 0.01	0.87 ± 0.04	1.03 ± 0.02	1.89 ± 0.03	1.28 ± 0.05	1.33 ± 0.01	0.74 ± 0.01	0.28 ± 0.01	1.41 ± 0.20

	Esters									
41	11,13-Dimethyltetradec-12-enyl acetate	N.d.	0.66 ± 0.01	N.d.	N.d.	N.d.	N.d.	N.d.	N.d.	N.d.
42	1-Nonyl-2-(prop-2-enyl) oxalate	N.d.	N.d.	N.d.	N.d.	N.d.	N.d.	N.d.	0.28 ± 0.01	0.41 ± 0.03
43	2-Methylbutyl acetate	0.45 ± 0.01	N.d.	N.d.	N.d.	N.d.	N.d.	0.57 ± 0.03	N.d.	N.d.
44	2-Phenylethyl acetate	0.24 ± 0.01	0.13 ± 0.01	0.17 ± 0.01	N.d.	0.14 ± 0.01	0.19 ± 0.01	0.19 ± 0.01	N.d.	N.d.
45	2-Tetradecyl 2-chloroacetate	0.15 ± 0.01	N.d.	N.d.	N.d.	N.d.	N.d.	N.d.	N.d.	N.d.
46	3-Tridecyl methoxyacetate	N.d.	0.55 ± 0.02	0.66 ± 0.05	0.42 ± 0.01	N.d.	N.d.	N.d.	1.42 ± 0.11	N.d.
47	Allyl pentadecyl oxalate	N.d.	N.d.	N.d.	N.d.	N.d.	N.d.	N.d.	N.d.	N.d.
48	Benzyl oleate	0.70 ± 0.05	N.d.	0.44 ± 0.03	0.17 ± 0.01	N.d.	N.d.	N.d.	N.d.	N.d.
49	Dodecan-2-yl prop-2-enoate	N.d.	N.d.	N.d.	N.d.	N.d.	N.d.	N.d.	N.d.	0.09 ± 0.01
50	Dodecyl propyl oxalate	N.d.	N.d.	N.d.	N.d.	N.d.	N.d.	N.d.	N.d.	0.27 ± 0.02
51	Ethyl 3-hydroxybutanoate	0.19 ± 0.01	0.04 ± 0.01	0.04 ± 0.00	0.06 ± 0.00	N.d.	0.04 ± 0.01	0.04 ± 0.00	N.d.	0.17 ± 0.08
52	Ethyl 3-phenylpropanoate	0.15 ± 0.01	N.d.	N.d.	N.d.	N.d.	N.d.	N.d.	N.d.	N.d.
53	Ethyl butanoate	N.d.	N.d.	N.d.	N.d.	N.d.	N.d.	N.d.	N.d.	0.19 ± 0.01
54	Ethyl decanoate	N.d.	N.d.	0.82 ± 0.02	0.69 ± 0.01	N.d.	0.55 ± 0.03	0.49 ± 0.01	N.d.	0.53 ± 0.04
55	Ethyl dodecanoate	N.d.	N.d.	0.38 ± 0.01	0.35 ± 0.01	N.d.	0.37 ± 0.01	0.28 ± 0.01	N.d.	0.15 ± 0.02
56	Ethyl hexadecanoate	N.d.	N.d.	0.37 ± 0.01	0.29 ± 0.01	N.d.	N.d.	N.d.	N.d.	N.d.
57	Ethyl hexanoate	1.04 ± 0.03	1.75 ± 0.02	0.46 ± 0.02	0.23 ± 0.01	N.d.	N.d.	0.14 ± 0.01	N.d.	N.d.
58	Ethyl octanoate	0.42 ± 0.01	0.10 ± 0.01	0.42 ± 0.03	0.53 ± 0.01	N.d.	0.43 ± 0.04	0.28 ± 0.04	N.d.	0.70 ± 0.24
59	Hexyl acetate	N.d.	N.d.	0.13 ± 0.01	N.d.	N.d.	N.d.	N.d.	N.d.	N.d.
60	Methyl benzoate	0.24 ± 0.01	N.d.	0.29 ± 0.01	N.d.	N.d.	N.d.	0.14 ± 0.01	N.d.	0.20 ± 0.01
61	Nonyl 2-acetyloxyacetate	N.d.	0.09 ± 0.00	N.d.	N.d.	N.d.	N.d.	N.d.	N.d.	N.d.
62	Octadecyl chloroacetate	0.24 ± 0.01	0.38 ± 0.01	0.43 ± 0.01	0.22 ± 0.01	N.d.	0.21 ± 0.01	0.20 ± 0.01	N.d.	0.34 ± 0.02
63	Pentyl 2,2-dimethylpropanoate	N.d.	N.d.	N.d.	N.d.	N.d.	N.d.	N.d.	N.d.	N.d.
64	Pentyl acetate	N.d.	0.05 ± 0.00	0.49 ± 0.02	N.d.	0.32 ± 0.01	0.43 ± 0.03	N.d.	N.d.	N.d.
	**Number of Esters**	10	9	13	9	2	7	9	2	10

	**Total concentration**	3.81 ± 0.05	3.75 ± 0.02	5.10 ± 0.05	2.97 ± 0.01	0.46 ± 0.01	2.21 ± 0.04	2.33 ± 0.04	1.70 ± 0.11	3.05 ± 0.24
	Furans									
65	2,5-Dimethyl-4-hydroxy-3(2H)-furanone	0.24 ± 0.01	0.21 ± 0.01	0.38 ± 0.01	0.37 ± 0.01	N.d.	0.29 ± 0.01	0.30 ± 0.01	0.31 ± 0.01	0.40 ± 0.02
66	4,4,5,5-Tetramethyl-dihydro-2(3H)-furanone	N.d.	N.d.	N.d.	N.d.	N.d.	0.15 ± 0.01	N.d.	N.d.	0.47 ± 0.18
	**Number of Furans**	1	1	1	1	N.d.	2	1	1	2
	**Total concentration**	0.24 ± 0.01	0.21 ± 0.01	0.38 ± 0.01	0.37 ± 0.01	N.d.	0.44 ± 0.01	0.30 ± 0.01	0.31 ± 0.01	0.87 ± 0.18
	Other compounds									
67	(Z)-3-Dodecene	N.d.	0.27 ± 0.01	N.d.	N.d.	0.65 ± 0.05	0.15 ± 0.01	N.d.	N.d.	N.d.
68	Nonadecane	N.d.	N.d.	N.d.	N.d.	0.93 ± 0.01	N.d.	N.d.	N.d.	N.d.
69	Tetradecane	0.97 ± 0.05	N.d.	2.06 ± 0.11	0.85 ± 0.02	0.33 ± 0.01	1.52 ± 0.06	1.30 ± 0.01	0.73 ± 0.03	1.90 ± 0.36
70	7-Tetradecene	N.d.	N.d.	N.d.	N.d.	0.68 ± 0.01	N.d.	N.d.	N.d.	N.d.
71	Hexadecane	N.d.	N.d.	2.51 ± 0.10	1.01 ± 0.08	N.d.	N.d.	N.d.	N.d.	N.d.
72	2-Methyl-2-pentyl-oxirane	N.d.	N.d.	N.d.	N.d.	N.d.	N.d.	N.d.	N.d.	N.d.
73	1-Decylsulfonyldecane	N.d.	N.d.	0.10 ± 0.01	N.d.	N.d.	N.d.	N.d.	N.d.	N.d.
74	Benzoyl bromide	0.73 ± 0.02	1.13 ± 0.01	1.15 ± 0.05	1.21 ± 0.03	3.60 ± 0.13	1.78 ± 0.08	1.21 ± 0.01	N.d.	1.94 ± 0.09
75	Methanesulfonyl azide	N.d.	N.d.	N.d.	N.d.	0.72 ± 0.01	N.d.	N.d.	N.d.	N.d.
76	Methoxyphenyl oxime	N.d.	N.d.	N.d.	N.d.	N.d.	N.d.	N.d.	N.d.	N.d.
77	O-Decyl-hydroxylamine	N.d.	N.d.	N.d.	N.d.	N.d.	N.d.	N.d.	N.d.	N.d.
78	n-Decyl sulfone	N.d.	N.d.	N.d.	N.d.	0.20 ± 0.01	N.d.	N.d.	N.d.	N.d.
79	1,2,3-Trimethylbenzene	N.d.	N.d.	N.d.	N.d.	N.d.	N.d.	N.d.	N.d.	N.d.
80	1,2,4-Trimethylbenzene	1.16 ± 0.13	0.69 ± 0.07	1.21 ± 0.05	1.22 ± 0.10	0.34 ± 0.09	1.99 ± 0.06	1.45 ± 0.05	1.01 ± 0.07	1.92 ± 0.40
81	2,4-Dimethyl-2-pentene	N.d.	N.d.	N.d.	N.d.	N.d.	N.d.	N.d.	N.d.	N.d.
82	3,5-Dimethyl-1-hexene	N.d.	N.d.	N.d.	N.d.	N.d.	N.d.	N.d.	N.d.	N.d.
	**Number of Other Compounds**	3	3	5	4	8	4	3	2	3
	**Total concentration**	2.86 ± 0.13	2.08 ± 0.07	7.03 ± 0.11	4.29 ± 0.10	7.45 ± 0.13	5.44 ± 0.08	3.96 ± 0.05	1.74 ± 0.07	5.77 ± 0.40

	**Number of Volatile Compounds**	23	29	35	32	15	28	29	20	36
	**Total concentration of Volatile Compounds**	19.75 ± 0.87	17.84 ± 0.62	21.86 ± 0.54	16.95 ± 0.37	16.93 ± 0.69	26.94 ± 0.96	17.57 ± 0.63	14.55 ± 0.41	25.28 ± 0.75

* N.d.: not detected.

Table S4. The results of the experimental design and analysis of the significance of the proposed model for total concentration of alcohols at 24 h of fermentation. Y: response (g/L); Ŷ: expected response (g/L); NS: non-significant coefficient; SS: sum of squares; df: degrees of freedom; QM: quadratic means; M: model; E: total error; Ee: experimental error; LF: lack of fit; Var(Ee): variance of the error experimental. SR: shaking rate; PMKG: proportion of milk kefir grains.

A	GW	Y	Ŷ	Coefficients	*t*	Model	
1	1	3.55	2.88	6.08	23.65	6.08	
1	–1	2.08	1.61	–1.00	4.61	–1.00	SR
–1	1	3.79	4.87	0.63	2.93	0.63	PMKG
–1	–1	2.35	3.60	0.00	0.03	NS	SR·PMKG
1.267	0	1.54	2.54	–1.42	5.54	–1.42	SR^2
–1.267	0	6.81	5.07	–1.42	5.57	–1.42	$PMKG^2$
0	1.267	4.82	4.60	Mean response = 4.50			
0	–1.267	3.51	2.99	Central mean response = 6.02			
0	0	5.42	6.08	Var(Ee) = 0.34			
0	0	5.72	6.08	t (α < 0.05; df = 4) = 2.78			
0	0	6.21	6.08	QMM/QME = 6.75		$F_8^4(\alpha = 0.05) = 3.84$	
0	0	6.92	6.08	QMLF/QMM = 0.63		$F_4^8(\alpha = 0.05) = 6.04$	
0	0	5.83	6.08	QME/QMEe = 3.39		$F_4^8(\alpha = 0.05) = 6.04$	
	SS	df	QM	QMLF/QMEe = 5.79		$F_4^4(\alpha = 0.05) = 6.39$	
Model	30.83	4	7.71	r^2 = 0.772		adjusted r^2 = 0.657	
Error	9.13	8	1.14				
Ee	1.35	4	0.34	Optimum SR = 69 rpm			
LF	7.79	4	1.95	Optimum PMKG = 3.30%			
Total	39.96	12	3.33	Maximum [Alcohols] = 6.32 mg/L			

Table S5. The results of the experimental design and analysis of the significance of the proposed model for total concentration of alcohols at 48 h of fermentation. Y: response (g/L); Ŷ: expected response (g/L); NS: non-significant coefficient; SS: sum of squares; df: degrees of freedom; QM: quadratic means; M: model; E: total error; Ee: experimental error; LF: lack of fit; Var(Ee): variance of the error experimental. SR: shaking rate; PMKG: proportion of milk kefir grains.

A	GW	Y	Ŷ	Coefficients	*t*	Model	
1	1	5.87	4.12	9.31	77.80	9.31	
1	–1	2.86	1.81	–1.37	13.65	–1.37	SR
–1	1	6.22	6.87	1.15	11.47	1.15	PMKG
–1	–1	2.08	4.56	–0.28	2.09	NS	SR·PMKG
1.267	0	2.42	4.65	–1.82	15.26	–1.82	SR^2
–1.267	0	10.57	8.13	–3.15	26.50	–3.15	$PMKG^2$
0	1.267	4.81	5.71	Mean response = 6.55			
0	–1.267	3.89	2.79	Central mean response = 9.29			
0	0	9.19	9.31	Var(Ee) = 0.07			
0	0	9.07	9.31	t ($\alpha < 0.05$; $df = 4$) = 2.78			
0	0	9.69	9.31	QMM/QME = 7.62		$F_8^4(\alpha = 0.05) = 3.84$	
0	0	9.43	9.31	QMLF/QMM = 0.63		$F_4^8(\alpha = 0.05) = 6.04$	
0	0	9.07	9.31	QME/QMEe = 41.10*		$F_4^8(\alpha = 0.05) = 6.04$	
	SS	df	QM	QMLF/QMEe = 81.20**		$F_4^4(\alpha = 0.05) = 6.39$	
Model	91.35	4	22.84	$r^2 = 0.792$		adjusted $r^2 = 0.688$	
Error	23.97	8	3.00				
Ee	0.29	4	0.07	Optimum SR***			
LF	23.68	4	5.92	Optimum PMKG***			
Total	115.32	12	9.61	Maximum [Alcohols]***			

* QME/QMEe > $F_4^8(\alpha = 0.05)$: the model is not statistically significant according to this criterion.

** QMLF/QMEe > $F_4^4(\alpha = 0.05)$: the model is not statistically significant according to this criterion.

*** Not calculated, as the model is not statistically significant based on the two criteria above.

Table S6. The results of the experimental design and analysis of the significance of the proposed model for total concentration of alcohols at 72 h of fermentation. Y: response (g/L); Ŷ: expected response (g/L); NS: non-significant coefficient; SS: sum of squares; df: degrees of freedom; QM: quadratic means; M: model; E: total error; Ee: experimental error; LF: lack of fit; Var(Ee): variance of the error experimental. SR: shaking rate; PMKG: proportion of milk kefir grains.

A	GW	Y	Ŷ	Coefficients	*t*	Model	
1	1	10.66	8.69	12.46	57.37	12.46	
1	–1	10.21	8.69	–0.61	3.35	–0.61	SR
–1	1	7.60	9.91	0.42	2.28	NS	PMKG
–1	–1	6.38	9.91	–0.19	0.79	NS	SR·PMKG
1.267	0	7.74	11.43	–0.46	2.12	NS	SR^2
–1.267	0	16.66	12.98	–2.90	13.45	–2.90	$PMKG^2$
0	1.267	8.80	7.54	Mean response = 10.59			
0	–1.267	7.75	7.54	Central mean response = 12.38			
0	0	11.73	12.20	Var(Ee) = 0.24			
0	0	13.04	12.20	t ($\alpha < 0.05$; $df = 4$) = 2.78			
0	0	12.43	12.20	QMM/QME = 4.28		$F_{10}^{2}(\alpha = 0.05) = 4.10$	
0	0	12.12	12.20	QMLF/QMM = 0.54		$F_{2}^{8}(\alpha = 0.05) = 19.37$	
0	0	12.58	12.20	QME/QMEe = 22.46*		$F_{4}^{10}(\alpha = 0.05) = 5.96$	
	SS	df	QM	QMLF/QMEe = 36.76**		$F_{4}^{6}(\alpha = 0.05) = 6.16$	
Model	46.11	2	23.06	$r^2 = 0.461$		adjusted $r^2 = 0.353$	
Error	53.92	10	5.39				
Ee	0.96	4	0.24	Optimum SR***			
LF	52.96	6	8.83	Optimum PMKG***			
Total	100.03	12	8.34	Maximum [Alcohols]***			

* QME/QMEe > $F_{4}^{10}(\alpha = 0.05)$: the model is not statistically significant according to this criterion.

** QMLF/QMEe > $F_{4}^{6}(\alpha = 0.05)$: the model is not statistically significant according to this criterion.

*** Not calculated, as the model is not statistically significant based on the two criteria above.

Table S7. The results of the experimental design and analysis of the significance of the proposed model for total concentration of aldehydes at 24 h of fermentation. Y: response (g/L); Ŷ: expected response (g/L); NS: non-significant coefficient; SS: sum of squares; df: degrees of freedom; QM: quadratic means; M: model; E: total error; Ee: experimental error; LF: lack of fit; Var(Ee): variance of the error experimental. SR: shaking rate; PMKG: proportion of milk kefir grains.

A	GW	Y	Ŷ	Coefficients	*t*	Model	
1	1	0.50	0.35	1.22	15.29	1.22	
1	–1	0.62	0.35	–0.50	7.41	–0.50	SR
–1	1	0.96	1.35	–0.01	0.18	NS	PMKG
–1	–1	0.56	1.35	–0.13	1.44	NS	SR·PMKG
1.267	0	0.00	0.54	–0.09	1.17	NS	SR^2
–1.267	0	2.51	1.80	–0.32	4.00	–0.32	$PMKG^2$
0	1.267	0.75	0.66	Mean response = 0.99			
0	–1.267	1.04	0.66	Central mean response = 1.19			
0	0	1.42	1.17	Var(Ee) = 0.03			
0	0	1.16	1.17	t ($\alpha < 0.05$; $df = 4$) = 2.78			
0	0	1.31	1.17	QMM/QME = 5.90	$F_{10}^{2}(\alpha = 0.05) = 4.10$		
0	0	1.07	1.17	QMLF/QMM = 0.45	$F_{2}^{8}(\alpha = 0.05) = 19.37$		
0	0	0.98	1.17	QME/QMEe = 6.01*	$F_{4}^{10}(\alpha = 0.05) = 5.96$		
	SS	df	QM	QMLF/QMEe = 9.34**	$F_{4}^{6}(\alpha = 0.05) = 6.16$		
Model	2.30	2	1.15	$r^2 = 0.541$	adjusted $r^2 = 0.450$		
Error	1.95	10	0.19				
Ee	0.13	4	0.03	Optimum SR***			
LF	1.82	6	0.30	Optimum PMKG***			
Total	4.24	12	0.35	Maximum [Aldehydes]***			

* QME/QMEe > $F_{4}^{10}(\alpha = 0.05)$: the model is not statistically significant according to this criterion.

** QMLF/QMEe > $F_{4}^{6}(\alpha = 0.05)$: the model is not statistically significant according to this criterion.

*** Not calculated, as the model is not statistically significant based on the two criteria above.

Table S8. The results of the experimental design and analysis of the significance of the proposed model for total concentration of aldehydes at 48 h of fermentation. Y: response (g/L); Ŷ: expected response (g/L); NS: non-significant coefficient; SS: sum of squares; df: degrees of freedom; QM: quadratic means; M: model; E: total error; Ee: experimental error; LF: lack of fit; Var(Ee): variance of the error experimental. SR: shaking rate; PMKG: proportion of milk kefir grains.

A	GW	Y	Ŷ	Coefficients	*t*	Model	
1	1	0.67	0.38	1.08	48.63	1.08	
1	–1	0.71	0.60	–0.43	22.97	–0.43	SR
–1	1	2.16	2.00	0.27	14.48	0.27	PMKG
–1	–1	0.67	0.70	–0.38	15.30	–0.38	SR·PMKG
1.267	0	0.00	0.27	–0.18	8.14	–0.18	SR^2
–1.267	0	1.29	1.35	0.04	1.76	NS	$PMKG^2$
0	1.267	1.19	1.44	Mean response = 1.00			
0	–1.267	0.80	0.76	Central mean response = 1.10			
0	0	1.10	1.10	Var(Ee) = 0.00			
0	0	1.13	1.10	t ($\alpha < 0.05$; $df = 4$) = 2.78			
0	0	1.16	1.10	QMM/QME = 19.00		$F_8^4(\alpha = 0.05) = 3.84$	
0	0	1.10	1.10	QMLF/QMM = 0.55		$F_4^8(\alpha = 0.05) = 6.04$	
0	0	1.02	1.10	QME/QMEe = 13.66*		$F_4^8(\alpha = 0.05) = 6.04$	
	SS	df	QM	QMLF/QMEe = 26.32**		$F_4^4(\alpha = 0.05) = 6.39$	
Model	2.59	4	0.65	$r^2 = 0.905$		adjusted $r^2 = 0.857$	
Error	0.27	8	0.03				
Ee	0.01	4	0.00	Optimum SR***			
LF	0.26	4	0.07	Optimum PMKG***			
Total	2.87	12	0.24	Maximum [Aldehydes]***			

* QME/QMEe > $F_4^8(\alpha = 0.05)$: the model is not statistically significant according to this criterion.

** QMLF/QMEe > $F_4^4(\alpha = 0.05)$: the model is not statistically significant according to this criterion.

*** Not calculated, as the model is not statistically significant based on the two criteria above.

Table S9. The results of the experimental design and analysis of the significance of the proposed model for total concentration of aldehydes at 72 h of fermentation. Y: response (g/L); Ŷ: expected response (g/L); NS: non-significant coefficient; SS: sum of squares; df: degrees of freedom; QM: quadratic means; M: model; E: total error; Ee: experimental error; LF: lack of fit; Var(Ee): variance of the error experimental. SR: shaking rate; PMKG: proportion of milk kefir grains.

A	GW	Y	Ŷ	Coefficients	*t*	Model	
1	1	1.33	0.97	1.82	34.22	1.82	
1	–1	0.71	0.95	–0.11	2.46	NS	SR
–1	1	0.71	0.49	–0.23	5.14	–0.23	PMKG
–1	–1	1.06	1.43	0.24	4.04	0.24	SR·PMKG
1.267	0	0.00	0.41	–0.91	17.18	–0.91	SR^2
–1.267	0	0.84	0.41	0.09	1.67	NS	$PMKG^2$
0	1.267	1.26	1.58	Mean response = 1.36			
0	–1.267	2.78	2.16	Central mean response = 1.81			
0	0	1.75	1.87	Var(Ee) = 0.01			
0	0	1.77	1.87	t (α < 0.05; df = 4) = 2.78			
0	0	2.02	1.87	QMM/QME = 11.36		$F_9^3(\alpha = 0.05) = 3.86$	
0	0	1.74	1.87	QMLF/QMM = 0.47		$F_3^8(\alpha = 0.05) = 8.85$	
0	0	1.77	1.87	QME/QMEe = 9.92*		$F_4^9(\alpha = 0.05) = 6.00$	
	SS	df	QM	QMLF/QMEe = 17.05**		$F_4^5(\alpha = 0.05) = 6.26$	
Model	4.87	3	1.62	r^2 = 0.791		adjusted r^2 = 0.721	
Error	1.29	9	0.14				
Ee	0.06	4	0.01	Optimum SR***			
LF	1.23	5	0.25	Optimum PMKG***			
Total	6.15	12	0.51	Maximum [Aldehydes]***			

* QME/QMEe > $F_4^9(\alpha = 0.05)$: the model is not statistically significant according to this criterion.

** QMLF/QMEe > $F_4^5(\alpha = 0.05)$: the model is not statistically significant according to this criterion.

*** Not calculated, as the model is not statistically significant based on the two criteria above.

Table S10. The results of the experimental design and analysis of the significance of the proposed model for total concentration of ketones at 24 h of fermentation. Y: response (g/L); Ŷ: expected response (g/L); NS: non-significant coefficient; SS: sum of squares; df: degrees of freedom; QM: quadratic means; M: model; E: total error; Ee: experimental error; LF: lack of fit; Var(Ee): variance of the error experimental. SR: shaking rate; PMKG: proportion of milk kefir grains.

A	GW	Y	Ŷ	Coefficients	*t*	Model	
1	1	1.06	1.18	1.89	14.24	1.89	
1	–1	0.62	1.18	–0.24	2.15	NS	SR
–1	1	1.46	1.18	0.09	0.83	NS	PMKG
–1	–1	0.98	1.18	–0.01	0.07	NS	SR·PMKG
1.267	0	1.02	1.72	–0.31	2.36	NS	SR^2
–1.267	0	1.79	1.72	–0.54	4.08	–0.54	$PMKG^2$
0	1.267	0.94	0.85	Mean response = 1.42			
0	–1.267	1.14	0.85	Central mean response = 1.89			
0	0	2.38	1.72	Var(Ee) = 0.09			
0	0	1.823	1.72	t (α < 0.05; df = 4) = 2.78			
0	0	1.923	1.72	QMM/QME = 10.74		$F_{11}^{1}(\alpha = 0.05) = 4.84$	
0	0	1.728	1.72	QMLF/QMM = 0.22		$F_{1}^{8}(\alpha = 0.05) = 238.88$	
0	0	1.595	1.72	QME/QMEe = 1.55		$F_{4}^{11}(\alpha = 0.05) = 5.94$	
	SS	df	QM	QMLF/QMEe = 1.87		$F_{4}^{7}(\alpha = 0.05) = 6.09$	
Model	4.87	3	1.62	r^2 = 0.494*		adjusted r^2 = 0.448**	
Error	1.29	9	0.14				
Ee	0.06	4	0.01	Optimum SR***			
LF	1.23	5	0.25	Optimum PMKG***			
Total	6.15	12	0.51	Maximum [Ketones]***			

* The value of r^2 is less than 0.500, indicating that the model has a considerably low predictive capability.

** The value of adjusted r^2 is less than 0.500, indicating that the model has a considerably low predictive capability.

*** Not calculated, as the model demonstrates low predictive capability.

Table S11. The results of the experimental design and analysis of the significance of the proposed model for total concentration of ketones at 48 h of fermentation. Y: response (g/L); Ŷ: expected response (g/L); NS: non-significant coefficient; SS: sum of squares; df: degrees of freedom; QM: quadratic means; M: model; E: total error; Ee: experimental error; LF: lack of fit; Var(Ee): variance of the error experimental. SR: shaking rate; PMKG: proportion of milk kefir grains.

A	GW	Y	Ŷ	Coefficients	t	Model	
1	1	0.64	0.88	0.85	47.93	0.85	
1	–1	0.46	0.62	–0.04	2.95	–0.04	SR
–1	1	0.41	0.45	–0.13	8.60	–0.13	PMKG
–1	–1	1.26	1.22	0.26	12.87	0.26	SR·PMKG
1.267	0	1.38	1.10	0.19	10.89	0.19	SR^2
–1.267	0	1.18	1.21	–0.25	14.21	–0.25	$PMKG^2$
0	1.267	0.47	0.29	Mean response = 0.82			
0	–1.267	0.67	0.61	Central mean response = 0.83			
0	0	0.78	0.85	Var(Ee) = 0.00			
0	0	0.83	0.85	t (α < 0.05; df = 4) = 2.78			
0	0	0.80	0.85	QMM/QME = 6.12		$F_7^5(\alpha = 0.05) = 3.97$	
0	0	0.87	0.85	QMLF/QMM = 0.76		$F_5^8(\alpha = 0.05) = 4.82$	
0	0	0.87	0.85	QME/QMEe = 18.61*		$F_4^7(\alpha = 0.05) = 4.88$	
	SS	df	QM	QMLF/QMEe = 42.08**		$F_4^3(\alpha = 0.05) = 6.59$	
Model	0.91	5	0.18	r^2 = 0.814		adjusted r^2 = 0.681	
Error	0.21	7	0.03				
Ee	0.01	4	0.00	Optimum SR***			
LF	0.20	3	0.07	Optimum PMKG***			
Total	1.12	12	0.09	Maximum [Ketones]***			

* QME/QMEe > $F_4^7(\alpha = 0.05)$: the model is not statistically significant according to this criterion.

** QMLF/QMEe > $F_4^3(\alpha = 0.05)$: the model is not statistically significant according to this criterion.

*** Not calculated, as the model is not statistically significant based on the two criteria above.

Table S12. The results of the experimental design and analysis of the significance of the proposed model for total concentration of ketones at 72 h of fermentation. Y: response (g/L); Ŷ: expected response (g/L); NS: non-significant coefficient; SS: sum of squares; df: degrees of freedom; QM: quadratic means; M: model; E: total error; Ee: experimental error; LF: lack of fit; Var(Ee): variance of the error experimental. SR: shaking rate; PMKG: proportion of milk kefir grains.

A	GW	Y	Ŷ	Coefficients	*t*	Model	
1	1	0.85	0.99	1.38	15.59	1.38	
1	–1	0.87	0.99	–0.18	2.35	NS	SR
–1	1	1.03	0.99	–0.04	0.55	NS	PMKG
–1	–1	1.89	0.99	0.21	2.10	NS	SR·PMKG
1.267	0	1.28	1.42	0.06	0.73	NS	SR^2
–1.267	0	1.33	1.42	–0.43	4.89	–0.43	$PMKG^2$
0	1.267	0.74	0.73	Mean response = 1.18			
0	–1.267	0.28	0.73	Central mean response = 1.41			
0	0	1.46	1.42	Var(Ee) = 0.04			
0	0	1.58	1.42	t (α < 0.05; df = 4) = 2.78			
0	0	1.43	1.42	QMM/QME = 8.51		$F_{11}^{1}(\alpha = 0.05) = 4.84$	
0	0	1.07	1.42	QMLF/QMM = 0.27		$F_{1}^{8}(\alpha = 0.05) = 238.88$	
0	0	1.51	1.42	QME/QMEe = 2.81		$F_{4}^{11}(\alpha = 0.05) = 5.94$	
	SS	df	QM	QMLF/QMEe = 3.84		$F_{4}^{7}(\alpha = 0.05) = 6.09$	
Model	0.96	1	0.96	r^2 = 0.436*		adjusted r^2 = 0.385**	
Error	1.24	11	0.11				
Ee	0.16	4	0.04	Optimum SR***			
LF	1.08	7	0.15	Optimum PMKG***			
Total	2.19	12	0.18	Maximum [Ketones]***			

* The value of r^2 is less than 0.500, indicating that the model has a considerably low predictive capability.

** The value of adjusted r^2 is less than 0.500, indicating that the model has a considerably low predictive capability.

*** Not calculated, as the model demonstrates low predictive capability.

Table S13. The results of the experimental design and analysis of the significance of the proposed model for total concentration of esters at 24 h of fermentation. Y: response (g/L); Ŷ: expected response (g/L); NS: non-significant coefficient; SS: sum of squares; df: degrees of freedom; QM: quadratic means; M: model; E: total error; Ee: experimental error; LF: lack of fit; Var(Ee): variance of the error experimental. SR: shaking rate; PMKG: proportion of milk kefir grains.

A	GW	Y	Ŷ	Coefficients	*t*	Model	
1	1	1.72	1.31	1.13	18.24	1.13	
1	–1	0.45	0.65	–0.53	10.22	–0.53	SR
–1	1	3.04	2.37	0.33	6.33	0.33	PMKG
–1	–1	1.64	1.71	–0.03	0.46	NS	SR·PMKG
1.267	0	0.95	1.06	0.37	5.95	0.37	SR^2
–1.267	0	2.00	2.40	0.02	0.29	NS	$PMKG^2$
0	1.267	0.80	1.56	Mean response = 1.34			
0	–1.267	1.03	0.72	Central mean response = 1.17			
0	0	1.21	1.14	Var(Ee) = 0.02			
0	0	1.35	1.14	t ($\alpha < 0.05$; $df = 4$) = 2.78			
0	0	1.19	1.14	QMM/QME = 6.64		$F_9^3(\alpha = 0.05) = 3.86$	
0	0	1.14	1.14	QMLF/QMM = 0.54		$F_3^8(\alpha = 0.05) = 8.85$	
0	0	0.97	1.14	QME/QMEe = 9.02*		$F_4^9(\alpha = 0.05) = 6.00$	
	SS	df	QM	QMLF/QMEe = 15.44**		$F_4^5(\alpha = 0.05) = 6.26$	
Model	3.52	3	1.17	$r^2 = 0.689$		adjusted $r^2 = 0.585$	
Error	1.59	9	0.18				
Ee	0.08	4	0.02	Optimum SR***			
LF	1.51	5	0.30	Optimum PMKG***			
Total	5.11	12	0.43	Maximum [Esters]***			

* QME/QMEe > $F_4^9(\alpha = 0.05)$: the model is not statistically significant according to this criterion.

** QMLF/QMEe > $F_4^5(\alpha = 0.05)$: the model is not statistically significant according to this criterion.

*** Not calculated, as the model is not statistically significant based on the two criteria above.

Table S14. The results of the experimental design and analysis of the significance of the proposed model for total concentration of esters at 48 h of fermentation. Y: response (g/L); Ŷ: expected response (g/L); NS: non-significant coefficient; SS: sum of squares; df: degrees of freedom; QM: quadratic means; M: model; E: total error; Ee: experimental error; LF: lack of fit; Var(Ee): variance of the error experimental. SR: shaking rate; PMKG: proportion of milk kefir grains.

A	GW	Y	Ŷ	Coefficients	*t*	Model	
1	1	2.01	1.24	1.75	65.76	1.75	
1	–1	0.81	0.57	–0.91	40.71	–0.91	SR
–1	1	2.60	3.06	0.34	15.17	0.34	PMKG
–1	–1	1.47	2.38	0.02	0.58	NS	SR·PMKG
1.267	0	0.43	1.26	0.41	15.63	0.41	SR^2
–1.267	0	4.62	3.56	–0.35	13.13	–0.35	$PMKG^2$
0	1.267	1.35	1.62	Mean response = 1.78			
0	–1.267	1.26	0.76	Central mean response = 1.73			
0	0	1.77	1.75	Var(Ee) = 0.00			
0	0	1.68	1.75	t (α < 0.05; df = 4) = 2.78			
0	0	1.79	1.75	QMM/QME = 4.32		$F_8^4(\alpha = 0.05) = 3.84$	
0	0	1.65	1.75	QMLF/QMM = 0.73		$F_4^8(\alpha = 0.05) = 6.04$	
0	0	1.75	1.75	QME/QMEe = 133.28*		$F_4^8(\alpha = 0.05) = 6.04$	
	SS	df	QM	QMLF/QMEe = 265.56**		$F_4^4(\alpha = 0.05) = 6.39$	
Model	8.29	4	2.07	r^2 = 0.684		adjusted r^2 = 0.525	
Error	3.84	8	0.48				
Ee	0.01	4	0.00	Optimum SR***			
LF	3.82	4	0.96	Optimum PMKG***			
Total	12.13	12	1.01	Maximum [Esters]***			

* QME/QMEe > $F_4^8(\alpha = 0.05)$: the model is not statistically significant according to this criterion.

** QMLF/QMEe > $F_4^4(\alpha = 0.05)$: the model is not statistically significant according to this criterion.

*** Not calculated, as the model is not statistically significant based on the two criteria above.

Table S15. The results of the experimental design and analysis of the significance of the proposed model for total concentration of esters at 72 h of fermentation. Y: response (g/L); Ŷ: expected response (g/L); NS: non-significant coefficient; SS: sum of squares; df: degrees of freedom; QM: quadratic means; M: model; E: total error; Ee: experimental error; LF: lack of fit; Var(Ee): variance of the error experimental. SR: shaking rate; PMKG: proportion of milk kefir grains.

A	GW	Y	Ŷ	Coefficients	*t*	Model	
1	1	3.81	2.41	2.86	26.86	2.86	
1	–1	3.75	2.62	–0.38	4.23	–0.38	SR
–1	1	5.10	4.20	0.41	4.64	0.41	PMKG
–1	–1	2.97	2.34	–0.52	4.31	–0.52	SR·PMKG
1.267	0	0.46	2.41	–0.18	1.70	NS	SR^2
–1.267	0	2.21	3.37	0.24	2.30	NS	$PMKG^2$
0	1.267	2.33	3.42	Mean response = 2.89			
0	–1.267	1.70	2.37	Central mean response = 3.05			
0	0	3.04	2.89	Var(Ee) = 0.06			
0	0	3.40	2.89	t (α < 0.05; df = 4) = 2.78			
0	0	3.02	2.89	QMM/QME = 0.86*		$F_9^3(\alpha = 0.05) = 3.86$	
0	0	2.73	2.89	QMLF/QMM = 1.65		$F_3^8(\alpha = 0.05) = 8.85$	
0	0	3.07	2.89	QME/QMEe = 22.36**		$F_4^9(\alpha = 0.05) = 6.00$	
	SS	df	QM	QMLF/QMEe = 39.45***		$F_4^5(\alpha = 0.05) = 6.26$	
Model	3.34	3	1.11	r^2 = 0.224****		adjusted r^2 = 0.035****	
Error	11.59	9	1.29				
Ee	0.23	4	0.06	Optimum SR*****			
LF	11.36	5	2.27	Optimum PMKG*****			
Total	14.93	12	1.24	Maximum [Esters]*****			

* QMM/QME < $F_9^3(\alpha = 0.05)$: the model is not statistically significant according to this criterion.

** QME/QMEe > $F_4^9(\alpha = 0.05)$: the model is not statistically significant according to this criterion.

*** QMLF/QMEe > $F_4^5(\alpha = 0.05)$: the model is not statistically significant according to this criterion.

**** The values of r^2 and adjusted r^2 are less than 0.500, indicating that the model has a considerably low predictive capability.

***** Not calculated, as the model is not statistically significant based on the two criteria above and the low predictive capability of the model.

Table S16. The results of the experimental design and analysis of the significance of the proposed model for total concentration of furans at 24 h of fermentation. Y: response (g/L); Ŷ: expected response (g/L); NS: non-significant coefficient; SS: sum of squares; df: degrees of freedom; QM: quadratic means; M: model; E: total error; Ee: experimental error; LF: lack of fit; Var(Ee): variance of the error experimental. SR: shaking rate; PMKG: proportion of milk kefir grains.

A	GW	Y	Ŷ	Coefficients	t	Model	
1	1	0.14	–0.03	0.07	2.16	NS	
1	–1	0.20	0.28	–0.46	17.83	–0.46	SR
–1	1	2.03	1.61	0.20	7.78	0.20	PMKG
–1	–1	0.66	0.49	–0.36	10.21	–0.36	SR·PMKG
1.267	0	0.00	0.02	0.33	10.85	0.33	SR^2
–1.267	0	0.79	1.19	0.19	6.10	0.19	$PMKG^2$
0	1.267	0.22	0.63	Mean response = 0.36			
0	–1.267	0.10	0.11	Central mean response = 0.10			
0	0	0.12	0.07	Var(Ee) = 0.00			
0	0	0.02	0.07	t (α < 0.05; df = 4) = 2.78			
0	0	0.13	0.07	QMM/QME = 10.63		$F_8^4(\alpha = 0.05) = 3.84$	
0	0	0.19	0.07	QMLF/QMM = 0.59		$F_4^8(\alpha = 0.05) = 6.04$	
0	0	0.04	0.07	QME/QMEe = 14.99*		$F_4^8(\alpha = 0.05) = 6.04$	
	SS	df	QM	QMLF/QMEe = 28.98**		$F_4^4(\alpha = 0.05) = 6.39$	
Model	3.12	4	0.78	r^2 = 0.842		adjusted r^2 = 0.762	
Error	0.59	8	0.07				
Ee	0.02	4	0.00	Optimum SR***			
LF	0.57	4	0.14	Optimum PMKG***			
Total	3.71	12	0.31	Maximum [Furans]***			

* QME/QMEe > $F_4^8(\alpha = 0.05)$: the model is not statistically significant according to this criterion.

** QMLF/QMEe > $F_4^4(\alpha = 0.05)$: the model is not statistically significant according to this criterion.

*** Not calculated, as the model is not statistically significant based on the two criteria above.

Table S17. The results of the experimental design and analysis of the significance of the proposed model for total concentration of furans at 48 h of fermentation. Y: response (g/L); Ŷ: expected response (g/L); NS: non-significant coefficient; SS: sum of squares; df: degrees of freedom; QM: quadratic means; M: model; E: total error; Ee: experimental error; LF: lack of fit; Var(Ee): variance of the error experimental. SR: shaking rate; PMKG: proportion of milk kefir grains.

A	GW	Y	Ŷ	Coefficients	*t*	Model	
1	1	0.10	0.02	0.51	22.95	0.51	
1	–1	0.16	0.10	–0.21	11.09	–0.21	SR
–1	1	0.58	0.62	0.05	2.81	0.05	PMKG
–1	–1	0.27	0.33	–0.09	3.70	–0.09	SR·PMKG
1.267	0	0.00	0.10	–0.09	4.07	–0.09	SR^2
–1.267	0	0.71	0.63	–0.15	6.76	–0.15	$PMKG^2$
0	1.267	0.31	0.34	Mean response = 0.38			
0	–1.267	0.21	0.20	Central mean response = 0.51			
0	0	0.52	0.51	Var(Ee) = 0.00			
0	0	0.43	0.51	t (α < 0.05; df = 4) = 2.78			
0	0	0.51	0.51	QMM/QME = 16.88	$F_7^5(\alpha = 0.05) = 3.97$		
0	0	0.52	0.51	QMLF/QMM = 0.66	$F_5^8(\alpha = 0.05) = 4.82$		
0	0	0.57	0.51	QME/QMEe = 2.45	$F_4^7(\alpha = 0.05) = 4.88$		
	SS	df	QM	QMLF/QMEe = 4.39	$F_4^3(\alpha = 0.05) = 6.59$		
Model	0.52	5	0.10	r^2 = 0.923	adjusted r^2 = 0.869		
Error	0.04	7	0.01				
Ee	0.01	4	0.00	Optimum SR = 31 rpm			
LF	0.03	3	0.01	Optimum PMKG = 3.23%			
Total	0.56	12	0.05	Maximum [Furans] = 0.65 mg/L			

Table S18. The results of the experimental design and analysis of the significance of the proposed model for total concentration of furans at 72 h of fermentation. Y: response (g/L); Ŷ: expected response (g/L); NS: non-significant coefficient; SS: sum of squares; df: degrees of freedom; QM: quadratic means; M: model; E: total error; Ee: experimental error; LF: lack of fit; Var(Ee): variance of the error experimental. SR: shaking rate; PMKG: proportion of milk kefir grains.

A	GW	Y	Ŷ	Coefficients	*t*	Model	
1	1	0.24	0.23	0.86	10.73	0.86	
1	–1	0.21	0.23	–0.12	1.77	NS	SR
–1	1	0.38	0.23	0.00	0.06	NS	PMKG
–1	–1	0.37	0.23	0.01	0.06	NS	SR·PMKG
1.267	0	0.00	0.31	–0.34	4.29	–0.34	SR^2
–1.267	0	0.44	0.31	–0.29	3.62	–0.29	$PMKG^2$
0	1.267	0.30	0.39	Mean response = 0.51			
0	–1.267	0.31	0.39	Central mean response = 0.87			
0	0	0.85	0.86	Var(Ee) = 0.03			
0	0	0.67	0.86	t (α < 0.05; df = 4) = 2.78			
0	0	0.83	0.86	QMM/QME = 16.87		$F_{10}^{2}(\alpha = 0.05) = 4.10$	
0	0	0.85	0.86	QMLF/QMM = 0.29		$F_{2}^{8}(\alpha = 0.05) = 19.37$	
0	0	1.16	0.86	QME/QMEe = 0.94		$F_{4}^{10}(\alpha = 0.05) = 5.96$	
	SS	df	QM	QMLF/QMEe = 0.89		$F_{4}^{6}(\alpha = 0.05) = 6.16$	
Model	1.02	2	0.51	r^2 = 0.771		adjusted r^2 = 0.726	
Error	0.30	10	0.03				
Ee	0.13	4	0.03	Optimum SR = 86 rpm			
LF	0.17	6	0.03	Optimum PMKG = 3.00%			
Total	1.33	12	0.11	Maximum [Furans] = 0.86 mg/L			

Table S19. The results of the experimental design and analysis of the significance of the proposed model for total concentration of "other compounds" at 24 h of fermentation. Y: response (g/L); Ŷ: expected response (g/L); NS: non-significant coefficient; SS: sum of squares; df: degrees of freedom; QM: quadratic means; M: model; E: total error; Ee: experimental error; LF: lack of fit; Var(Ee): variance of the error experimental. SR: shaking rate; PMKG: proportion of milk kefir grains.

A	GW	Y	Ŷ	Coefficients	*t*	Model	
1	1	3.74	4.70	4.93	18.24	4.93	
1	–1	2.15	3.22	–0.58	2.54	NS	SR
–1	1	5.65	4.70	0.74	3.27	0.74	PMKG
–1	–1	3.93	3.22	–0.03	0.11	NS	SR·PMKG
1.267	0	6.15	5.26	0.60	2.22	NS	SR^2
–1.267	0	6.52	5.26	–1.30	4.83	–1.30	$PMKG^2$
0	1.267	4.10	4.12	Mean response = 4.54			
0	–1.267	2.49	2.24	Central mean response = 4.86			
0	0	4.87	5.26	Var(Ee) = 0.37			
0	0	4.01	5.26	t (α < 0.05; df = 4) = 2.78			
0	0	4.86	5.26	QMM/QME = 7.71		$F_{10}^{2}(\alpha = 0.05) = 4.10$	
0	0	4.81	5.26	QMLF/QMM = 0.38		$F_{2}^{8}(\alpha = 0.05) = 19.37$	
0	0	5.74	5.26	QME/QMEe = 2.20		$F_{4}^{10}(\alpha = 0.05) = 5.96$	
	SS	df	QM	QMLF/QMEe = 3.01		$F_{4}^{6}(\alpha = 0.05) = 6.16$	
Model	12.66	2	6.33	r^2 = 0.607		adjusted r^2 = 0.528	
Error	8.21	10	0.82				
Ee	1.49	4	0.37	Optimum SR = Did not affect the production of this VOC			
LF	6.72	6	1.12	Optimum PMKG = 3.38%			
Total	20.87	12	1.74	Maximum [Other compounds] = 5.04 mg/L			

Table S20. The results of the experimental design and analysis of the significance of the proposed model for total concentration of “other compounds” at 48 h of fermentation. Y: response (g/L); Ŷ: expected response (g/L); NS: non-significant coefficient; SS: sum of squares; df: degrees of freedom; QM: quadratic means; M: model; E: total error; Ee: experimental error; LF: lack of fit; Var(Ee): variance of the error experimental. SR: shaking rate; PMKG: proportion of milk kefir grains.

A	GW	Y	Ŷ	Coefficients	*t*	Model	
1	1	2.26	3.85	4.30	24.24	4.30	
1	–1	1.24	1.59	0.12	0.82	NS	SR
–1	1	1.42	1.59	–0.30	1.99	NS	PMKG
–1	–1	4.92	3.85	1.13	5.65	1.13	SR·PMKG
1.267	0	5.94	4.26	–0.07	0.41	NS	SR^2
–1.267	0	3.00	4.26	–1.53	8.70	–1.30	$PMKG^2$
0	1.267	2.26	1.80	Mean response = 3.41			
0	–1.267	1.99	1.80	Central mean response = 4.25			
0	0	4.33	4.26	Var(Ee) = 0.16			
0	0	3.61	4.26	t (α < 0.05; df = 4) = 2.78			
0	0	4.32	4.26	QMM/QME = 9.42		$F_{10}^{2}(\alpha = 0.05) = 4.10$	
0	0	4.28	4.26	QMLF/QMM = 0.37		$F_{2}^{8}(\alpha = 0.05) = 19.37$	
0	0	4.72	4.26	QME/QMEe = 5.71		$F_{4}^{10}(\alpha = 0.05) = 5.96$	
	SS	df	QM	QMLF/QMEe = 8.85*		$F_{4}^{6}(\alpha = 0.05) = 6.16$	
Model	17.21	2	8.60	r^2 = 0.653		adjusted r^2 = 0.584	
Error	9.14	10	0.91				
Ee	0.64	4	0.16	Optimum SR**			
LF	8.50	6	1.42	Optimum PMKG**			
Total	26.35	12	2.20	Maximum [Other compounds]**			

* QMLF/QMEe > $F_{4}^{6}(\alpha = 0.05)$: the model is not statistically significant according to this criterion.

** Not calculated, as the model is not statistically significant based on the two criteria above.

Table S21. The results of the experimental design and analysis of the significance of the proposed model for total concentration of "other compounds" at 72 h of fermentation. Y: response (g/L); Ŷ: expected response (g/L); NS: non-significant coefficient; SS: sum of squares; df: degrees of freedom; QM: quadratic means; M: model; E: total error; Ee: experimental error; LF: lack of fit; Var(Ee): variance of the error experimental. SR: shaking rate; PMKG: proportion of milk kefir grains.

A	GW	Y	Ŷ	Coefficients	*t*	Model	
1	1	2.86	4.39	5.79	32.69	5.79	
1	–1	2.08	2.63	–0.53	3.57	–0.53	SR
–1	1	7.03	5.45	0.88	5.90	0.88	PMKG
–1	–1	4.29	3.70	–0.49	2.45	NS	SR·PMKG
1.267	0	7.45	5.29	0.31	1.79	NS	SR^2
–1.267	0	5.44	6.64	–1.92	10.93	–1.92	$PMKG^2$
0	1.267	3.96	3.99	Mean response = 4.90			
0	–1.267	1.74	1.76	Central mean response = 5.77			
0	0	5.73	5.97	Var(Ee) = 0.16			
0	0	5.29	5.97	t (α < 0.05; df = 4) = 2.78			
0	0	5.70	5.97	QMM/QME = 6.45	$F_9^3(\alpha = 0.05) = 3.86$		
0	0	5.73	5.97	QMLF/QMM = 0.54	$F_3^8(\alpha = 0.05) = 8.85$		
0	0	6.40	5.97	QME/QMEe = 8.62*	$F_4^9(\alpha = 0.05) = 6.00$		
	SS	df	QM	QMLF/QMEe = 14.72**	$F_4^5(\alpha = 0.05) = 6.26$		
Model	26.70	3	8.90	r^2 = 0.683	adjusted r^2 = 0.577		
Error	12.41	9	1.38				
Ee	0.64	4	0.16	Optimum SR***			
LF	11.77	5	2.35	Optimum PMKG***			
Total	39.11	12	3.26	Maximum [Other compounds]***			

* QME/QMEe > $F_4^9(\alpha = 0.05)$: the model is not statistically significant according to this criterion.

** QMLF/QMEe > $F_4^5(\alpha = 0.05)$: the model is not statistically significant according to this criterion.

*** Not calculated, as the model is not statistically significant based on the two criteria above.

Table S22. The results of the experimental design and analysis of the significance of the proposed model for total concentration of volatile compounds ([TVOCs]) at 24 h of fermentation. Y: response (g/L); Ŷ: expected response (g/L); NS: non-significant coefficient; SS: sum of squares; df: degrees of freedom; QM: quadratic means; M: model; E: total error; Ee: experimental error; LF: lack of fit; Var(Ee): variance of the error experimental. SR: shaking rate; PMKG: proportion of milk kefir grains.

A	GW	Y	Ŷ	Coefficients	*t*	Model	
1	1	11.06	10.26	13.92	16.98	13.92	
1	–1	6.12	6.19	–3.25	4.72	–3.25	SR
–1	1	16.94	16.77	2.04	2.95	2.04	PMKG
–1	–1	10.07	12.70	–0.48	0.52	NS	SR·PMKG
1.267	0	9.66	9.95	0.27	0.33	NS	SR^2
–1.267	0	20.42	18.19	–2.59	3.18	–2.59	$PMKG^2$
0	1.267	11.59	12.49	Mean response = 12.63			
0	–1.267	9.32	7.33	Central mean response = 13.81			
0	0	16.95	14.07	Var(Ee) = 3.42			
0	0	12.58	14.07	t (α < 0.05; df = 4) = 2.78			
0	0	13.05	14.07	QMM/QME = 13.45		$F_9^3(\alpha = 0.05) = 3.86$	
0	0	12.50	14.07	QMLF/QMM = 0.42		$F_3^8(\alpha = 0.05) = 8.85$	
0	0	13.99	14.07	QME/QMEe = 1.02		$F_4^9(\alpha = 0.05) = 6.00$	
	SS	df	QM	QMLF/QMEe = 1.03		$F_4^5(\alpha = 0.05) = 6.26$	
Model	126.65	3	42.22	r^2 = 0.803		adjusted r^2 = 0.738	
Error	30.97	9	3.44				
Ee	3.67	4	0.92	Optimum SR = 25 rpm			
LF	27.30	5	5.46	Optimum PMKG = 3.52%			
Total	157.62	12	13.14	Maximum [TVOCs] = 18.55 mg/L			

Table S23. The results of the experimental design and analysis of the significance of the proposed model for total concentration of volatile compounds ([TVOCs]) at 48 h of fermentation. Y: response (g/L); Ŷ: expected response (g/L); NS: non-significant coefficient; SS: sum of squares; df: degrees of freedom; QM: quadratic means; M: model; E: total error; Ee: experimental error; LF: lack of fit; Var(Ee): variance of the error experimental. SR: shaking rate; PMKG: proportion of milk kefir grains.

A	GW	Y	Ŷ	Coefficients	*t*	Model	
1	1	11.56	9.51	13.86	32.65	13.86	
1	–1	6.23	6.71	–2.84	7.96	–2.84	SR
–1	1	13.41	15.19	1.40	3.93	1.40	PMKG
–1	–1	10.67	12.39	0.65	1.35	NS	SR·PMKG
1.267	0	10.17	10.60	0.61	1.44	NS	SR^2
–1.267	0	21.37	17.79	–3.25	7.69	–3.25	$PMKG^2$
0	1.267	10.39	10.76	Mean response = 12.39			
0	–1.267	8.78	7.21	Central mean response = 13.71			
0	0	14.38	14.20	Var(Ee) = 0.92			
0	0	14.00	14.20	t ($\alpha < 0.05$; $df = 4$) = 2.78			
0	0	14.19	14.20	QMM/QME = 12.27	$F_9^3(\alpha = 0.05) = 3.86$		
0	0	12.02	14.20	QMLF/QMM = 0.46	$F_3^8(\alpha = 0.05) = 8.85$		
0	0	13.95	14.20	QME/QMEe = 3.75	$F_4^9(\alpha = 0.05) = 6.00$		
	SS	df	QM	QMLF/QMEe = 5.95	$F_4^5(\alpha = 0.05) = 6.26$		
Model	126.65	3	42.22	$r^2 = 0.803$	adjusted $r^2 = 0.738$		
Error	30.97	9	3.44				
Ee	3.67	4	0.92	Optimum SR = 25 rpm			
LF	27.30	5	5.46	Optimum PMKG = 3.29%			
Total	157.62	12	13.14	Maximum [TVOCs] = 17.70 mg/L			

Table S24. The results of the experimental design and analysis of the significance of the proposed model for total concentration of volatile compounds ([TVOCs]) at 72 h of fermentation. Y: response (g/L); Ŷ: expected response (g/L); NS: non-significant coefficient; SS: sum of squares; df: degrees of freedom; QM: quadratic means; M: model; E: total error; Ee: experimental error; LF: lack of fit; Var(Ee): variance of the error experimental. SR: shaking rate; PMKG: proportion of milk kefir grains.

A	GW	Y	Ŷ	Coefficients	*t*	Model	
1	1	19.75	18.61	24.60	49.48	24.60	
1	–1	17.84	15.65	–1.93	4.61	–1.93	SR
–1	1	21.86	22.46	1.48	3.53	1.48	PMKG
–1	–1	16.95	19.51	–0.75	1.34	NS	SR·PMKG
1.267	0	16.93	21.49	–1.21	2.45	NS	SR^2
–1.267	0	26.94	26.37	–4.87	9.85	–4.87	$PMKG^2$
0	1.267	17.57	17.98	Mean response = 21.23			
0	–1.267	14.55	14.24	Central mean response = 24.72			
0	0	25.28	23.93	Var(Ee) = 1.26			
0	0	25.13	23.93	t (α < 0.05; df = 4) = 2.78			
0	0	25.92	23.93	QMM/QME = 11.64		$F_9^3(\alpha = 0.05) = 3.86$	
0	0	24.24	23.93	QMLF/QMM = 0.46		$F_3^8(\alpha = 0.05) = 8.85$	
0	0	23.02	23.93	QME/QMEe = 3.75		$F_4^9(\alpha = 0.05) = 6.00$	
	SS	df	QM	QMLF/QMEe = 5.94		$F_4^5(\alpha = 0.05) = 6.26$	
Model	164.81	3	54.94	r^2 = 0.795		adjusted r^2 = 0.727	
Error	42.47	9	4.72				
Ee	5.04	4	1.26	Optimum SR = 25 rpm			
LF	37.43	5	7.49	Optimum PMKG = 3.20%			
Total	207.28	12	17.27	Maximum [TVOCs] = 27.22 mg/L			

Table S25. Total variance explained by the principal component analysis (PCA) of the 28 kiwifruit-based beverages, based on the initial concentrations of each VOC.

	Initial Eigenvalues			Extraction Sums of Squared Loadings		
Component	Total	% of Variance	Cumulative %	Total	% of Variance	Cumulative %
1	11.162	13.612	13.612	11.162	13.612	13.612
2	7.504	9.151	22.764	7.504	9.151	22.764
3	6.628	8.083	30.846	6.628	8.083	30.846
4	5.760	7.024	37.871	5.760	7.024	37.871
5	5.418	6.607	44.478	5.418	6.607	44.478
6	5.107	6.228	50.705	5.107	6.228	50.705
7	4.617	5.631	56.336	4.617	5.631	56.336
8	4.375	5.335	61.671	4.375	5.335	61.671
9	3.428	4.181	65.852	3.428	4.181	65.852
10	3.259	3.975	69.827	3.259	3.975	69.827
11	3.104	3.785	73.612	3.104	3.785	73.612
12	2.680	3.269	76.880	2.680	3.269	76.880
13	2.378	2.899	79.780	2.378	2.899	79.780
14	2.335	2.848	82.628	2.335	2.848	82.628
15	2.140	2.609	85.237	2.140	2.609	85.237
16	1.934	2.358	87.595	1.934	2.358	87.595
17	1.480	1.805	89.401	1.480	1.805	89.401
18	1.429	1.742	91.143	1.429	1.742	91.143
19	1.385	1.689	92.831	1.385	1.689	92.831
20	1.241	1.513	94.344	1.241	1.513	94.344
21	1.140	1.390	95.734	1.140	1.390	95.734

Extraction meyhod: Principal component analysis.

Table S26. Total variance explained by principal component analysis (PCA) of the 28 kiwifruit-based beverages, based on the OAVs of VOCs with OAV ≥ 1.0.

	Initial Eigenvalues			Extraction Sums of Squared Loadings		
Component	Total	% of Variance	Cumulative %	Total	% of Variance	Cumulative %
1	6.665	17.539	17.539	6.665	17.539	17.539
2	4.852	12.768	30.308	4.852	12.768	30.308
3	4.195	11.039	41.346	4.195	11.039	41.346
4	3.522	9.269	50.616	3.522	9.269	50.616
5	2.936	7.727	58.342	2.936	7.727	58.342
6	2.421	6.372	64.714	2.421	6.372	64.714
7	2.084	5.485	70.200	2.084	5.485	70.200
8	1.776	4.673	74.873	1.776	4.673	74.873
9	1.566	4.122	78.994	1.566	4.122	78.994
10	1.447	3.807	82.802	1.447	3.807	82.802
11	1.145	3.012	85.814	1.145	3.012	85.814
12	1.026	2.700	88.514	1.026	2.700	88.514

Extraction meyhod: Principal component analysis.

Table S27. Total variance explained by principal component analysis (PCA) of the 28 kiwifruit-based beverages, based on microbiological, chemical, and aromatic characteristics.

	Initial Eigenvalues			Extraction Sums of Squared Loadings		
Component	Total	% of Variance	Cumulative %	Total	% of Variance	Cumulative %
1	3.994	36.310	36.310	3.994	36.310	36.310
2	3.289	29.897	66.207	3.289	29.897	66.207
3	1.760	15.996	82.203	1.760	15.996	82.203

Extraction meyhod: Principal component analysis.
